# The function of TLR2 and the microbiome in macrophage-dependent dissemination of nontuberculous mycobacterial gut infection

**DOI:** 10.7150/ijbs.124776

**Published:** 2026-05-18

**Authors:** Li Liu, Wanbin Hu, Herman P. Spaink

**Affiliations:** Institute of Biology Leiden, Animal Science and Health, Leiden University, Einsteinweg 55, 2333 CC Leiden, The Netherlands.

**Keywords:** Nontuberculous mycobacteria, TLR2, microbiome, zebrafish, natural immersion infection

## Abstract

**Background:**

Nontuberculous mycobacteria (NTM) infections are increasing in incidence and mortality worldwide, yet the *in vivo* determinants of early intestinal colonization and subsequent dissemination remain poorly defined. Using a zebrafish larval model, we studied dissemination of two NTM strains, *Mycobacterium avium* subspecies* hominissuis Chester* (strain MAC 101) and *M. marinum* (strain Mma20) through the gastrointestinal (GI) tract. We performed bacterial immersion experiments and gut microinjection with these pathogens using *tlr2* mutant and wild-type zebrafish larvae in both germ-free (GF) and microbiome-colonized conditions. This study for the first time shows the roles of Toll-like receptor 2 (TLR2) and the microbiome in orchestrating immune responses in NTM gut infection and dissemination.

**Results:**

In wild-type microbiome-colonized larvae, MAC 101 predominantly localizes in the posterior gut, in contrast to an anterior-biased distribution for Mma20 after 2.5 days of immersion infection. Both MAC 101 and Mma20 disseminate to posterior body region after 2.5 days of immersion infection. Robotic gut microinjection confirms the protective roles of TLR2 and the microbiome against proliferation of MAC 101 and Mma20. Expression analysis of downstream genes indicate that patterns of TLR2-dependent gene regulation differ between the two NTM species and show that the presence or absence of the microbiome differentially influences specific transcriptional responses to infection. Macrophage ablation studies show that macrophages facilitate dissemination of gut bacteria to the posterior body region. Quantification of macrophages containing bacteria throughout the body show that the dissemination of bacteria by macrophages depends on TLR2, but not on the microbiome. Using the same approach, TLR2 chemical antagonist treatment confirms the results observed in *tlr2* mutant larvae. Live imaging of macrophage trajectories after bacterial gut microinjection show that macrophage motility after infection is impaired in *tlr2* mutant larvae compared to the wild type. Notably, the effect of TLR2 on macrophage motility differs between GF and microbiome-colonized conditions.

**Conclusions:**

TLR2 and the microbiome play critical roles in modulating host responses to MAC 101 and Mma20 gut infection. Our findings provide new insights into the coordinated roles of TLR2 signaling and the microbiome in controlling infection of mycobacteria via the gut and underscore the importance of TLR2 in macrophage function during mycobacterial gut infection and dissemination.

## 1. Introduction

Host innate immunity is triggered by evolutionarily conserved pattern recognition receptors (PRRs), such as Toll-like receptors (TLRs), which detect pathogen-associated molecular patterns (PAMPs) from invading bacteria, viruses and other pathogens 1, 2. Toll-like receptor 2 (TLR2) is a key PRR that recognizes various microbial components, including lipoproteins and glycolipids 3, 4. Upon PAMP recognition, TLR2 triggers downstream signaling cascades that lead innate immune cells to secrete inflammatory cytokines through mitogen-activated protein kinase (MAPK)/Nuclear factor kappa-light-chain-enhancer of activated B cells (NF-κB) signaling pathways 5-7. TLR2 has been shown to play a critical role in initiating host innate immune responses against *Mycobacterium tuberculosis* and other mycobacterial species outside the *M. tuberculosis* complex collectively called nontuberculous mycobacteria (NTM) 8-10. Nowadays, NTM infectious diseases are increasing in incidence, prevalence and mortality globally 11-13. Therefore, understanding the host immune mechanisms involved in NTM infections, including the role of pattern recognition receptors like TLR2, is essential for developing effective host-directed therapies for prevention and treatment of NTM infectious diseases.

The structural integrity of TLR2 is essential for host defense against pathogens, and single nucleotide polymorphisms in the human TLR2 have been linked to the increased susceptibility to mycobacterial infectious diseases 14-17. Furthermore, several studies on mycobacterial infection in animal models, including rodents and zebrafish, have shown that TLR2, in concert with other TLRs, plays a protective role in host defense 18-21. In these studies, mycobacteria were infected through direct lung aerosol infection, intranasal, intravenous, or intratracheal infection methods. However, the role of TLR2 in defense against gut mycobacterial infectious diseases infected via the gastrointestinal (GI) tract is less known 22. Most studies focused on the function of TLR2 in Johne's disease caused by intestinal infection by *Mycobacterium avium* (*M. avium*) subspecies *paratuberculosis* (MAP) in ruminants and many other farm animals 23, 24. In a previous murine MAP oral exposure study, an increased colonic expression of TLR2 upon oral-gut MAP infection was found 23. For ovine Johne's disease in Turkish sheep, the TLR2 haplotypes encoding Q650 was clarified to facilitate selective breeding for sheep with reduced susceptibility to ovine Johne's disease 24. Some studies have shown that MAP infection is a potential causative factor in human Crohn's disease, a chronic inflammatory disease of the GI tract 25-28. However, the role of TLR2 in mycobacterial infection of the human gut has been poorly documented.

The zebrafish (*Danio rerio*) larval model is a well-established animal model for studying host-pathogen interactions and innate immunity *in vivo* 29, 30. In previous studies, we have established a NTM infectious model in zebrafish larvae and found that TLR2 has a function in response to *M. avium* infection after both bacterial caudal blood island injection and tail fin injection 15, 31. For bacterial gut infection studies, zebrafish larvae are also a powerful animal model due to their optical transparency that enable real-time, non-invasive imaging of bacterial localization and dissemination and immune cell behavior 32. Versatile gnotobiotic zebrafish techniques, such as the generation of germ-free (GF) larvae, make zebrafish an ideal model to study the function of microbiome 33-35. The zebrafish gut contains functionally relevant epithelial cells and mucus-producing goblet cells, closely resembling the mammalian intestinal barrier 36. Additionally, the zebrafish microbiota has been shown to influence gut immunity in ways comparable to mammals 33, 37. A recent study has shown that intestinal microbial colonization modulates host immune states in zebrafish through a TLR2-Myd88-dependent signaling pathway 38. In a multitude of studies, the microbiome has been shown to have a critical role in controlling gut infections by various pathogens 39, 40. However, the influence of the microbiome on mycobacterial gut infection remains unexplored.

In this study, we used zebrafish larvae to investigate the roles of TLR2 and the microbiome in modulating immune responses to gut infections with two NTM species. *M. avium* complex (MAC), consisting of *M. avium* and *M. intracellulare*, is one of the most commonly encountered NTM species groups associated with human disease 41-43. We used the strain *M. avium* subspecies* hominissuis Chester* (MAC 101). MAC 101 is a well-characterized reference strain that is widely used in experimental settings 15. We selected MAC 101 over other members of the *M. avium* complex, such as MAP, for both biological and practical reasons. MAP has an exceptionally slow growth rate and requires prolonged culture, which is incompatible with the rapid developmental timeline of zebrafish larvae and the time window accessible for larval infection assays. The use of a genetically well characterized, commercially available reference strain that also has been tested in mammalian studies further facilitates reproducibility and comparison across studies 44. *M. marinum*, a natural pathogen of ectothermic animals such as fish, amphibians, and reptiles, shares key virulence factors with *M. tuberculosis* and can also cause granulomatous skin infections in humans 45. By employing both natural immersion infection and targeted gut microinjection with MAC 101 and Mma20, we examined bacterial dissemination, transcriptional responses and macrophage behavior under germ-free (GF) and microbiome-colonized (CONVD) conditions in *tlr2* mutant and wild-type larvae. Our results show that MAC 101 and Mma20 exhibit distinct gut localization patterns depending on the microbiome and TLR2. We show the function of macrophages in dissemination of mycobacterial infection to the posterior body region. We therefore performed quantitative, dynamic imaging studies investigating the roles of the microbiome and TLR2 in macrophage-based dissemination of mycobacteria from the gut, as well as their migratory behavior toward the gut and during subsequent dissemination of infection. Our findings for the first time provide new insights into the interplay between host innate sensors and the microbiome in the context of mycobacterial gut pathogenesis.

## 2. Materials and Methods

### 2.1. Zebrafish maintenance and mutant line construction

The maintenance of all adult and larval zebrafish and all animal experiments described in this study were conducted at Leiden University in accordance with the standard protocols (zfin.org) and adhered to the international guidelines specified by the EU Animal Protection Directive 2010/63/EU. The culture of adult fish was approved by the university's local animal welfare committee (DEC) (License number: protocol 14,198) and no adult zebrafish were sacrificed in this study. Eggs and larvae were grown in laboratory-manufactured egg water (containing 60 mg/l instant ocean sea salts) at 28℃. For immersion infection, gut injection and live imaging assays, the experiments were all performed on larvae up to 5 days post fertilization (dpf) and therefore prior to the free-feeding stage, which is not covered by the animal experimentation law according to the EU Animal Protection Directive 2010/63/EU. 4 dpf larvae during gut injection experiments were anesthetized with egg water containing 0.02% buffered 3-aminobenzoic acid ethyl ester (Tricaine, Sigma-Aldrich, Netherlands).

The *tlr2^sa19423^* mutant (further referred as *tlr2^-/-^* or *tlr2* mutant) line was used in this study. This mutant line was identified from an ENU-mutagenized zebrafish library by sequencing (ZFIN Cat# ZDB-ALT-131217-14694, RRID: ZFIN_ZDB-ALT-131217-14694) 31. All mutant alleles were identified by sequencing, and homozygote mutant carriers were outcrossed more than three generations to AB/TL wild-type line. Homozygote mutants and their wild-type siblings, hereafter referred as *tlr2^+/+^*, were used in this study. Therefore, the *tlr2* mutant and wild-type fish lines used in this study have an AB/TL background. To investigate the effect of the *tlr2* mutation on macrophage function and behavior, fluorescent transgenic lines *tlr2^+/+^ Tg* (*mpeg1*:* EGFP)^gl22^* and *tlr2^-/-^ Tg* (*mpeg1*:* EGFP*)*^gl22^* were used. The generation of these transgenic lines refers to previous studies 46. For macrophage ablation experiments, *Tg* (*mpeg:Gal4^gl25^; UAS:NTR-mCherry^c264^*) fish line was used 47.

### 2.2. Bacterial strain preparation

Two types of NTM strains, *M. avium* subspecies* hominissuis Chester* (MAC 101; ATCC, 700898^TM^; genome sequence deposited in GenBank: CP195520.1) and* M. marinum* m20 (further referred as* M. marinum* or Mma20), both expressing red fluorescent protein (MAC 101: mCherry; Mma20: DsRed), were used to infect zebrafish larvae in this study 15, 48. In addition, we used a MAC 101 derivative that expresses mWasabi to perform the co-infection of MAC 101 and Mma20 15. The MAC 101 mCherry and mWasabi strains were grown at 37°C and the Mma20 DsRed strain was grown at 28 °C in Middlebrook 7H9 broth (Fort Richard) supplemented with 10% acid-albumin-dextrose-catalase (ADC) enrichment medium (Fort Richard) and 50 µg/mL of hygromycin for selection of fluorescent.

### 2.3. Germ-free larvae generation

Germ-free (GF) and conventionalized (CONVD) embryos were generated using the previously described “Natural breeding method” with several modifications 34, 38. Briefly, all embryos were divided into two groups, one group was treated with antibiotic mix, Ampicillin (250 µg/ml), Kanamycin (5 µg/ml) and amphotericin B (250 ng/ml) after the harvest of embryos and maintained in autoclaved sterile egg water. At 6 hours post fertilization (hpf), all embryos from this group were washed by using 0.2 % Povidone-Iodine (PVP-I) and 0.003% bleach to process the sterilization. Another group was maintained in a non-sterile egg water (without any treatment, as conventionally reared group). At 2.5 dpf (60 hpf), half of the sterilized larvae were randomly assigned to microbial conventionalization by transferring them into water from petri dishes of the conventionally reared group, generating the CONVD group. The remaining sterilized larvae were maintained in sterile egg water as the GF group. Only morphologically normal larvae were used for subsequent analyses.

Sterility of the GF group was monitored by plating 2 ml of water from GF group petri dishes on Luria-Bertani (LB) agar plates and incubating the plates under aerobic conditions at 28 °C for 2 days.

### 2.4. Natural immersion infection

In the present study, mCherry-labeled MAC 101 and DsRed-labeled Mma20 bacteria were used for natural immersion infection experiments. Bacterial inocula were prepared as previously described 49. Prior to infection, MAC 101 and Mma20 bacteria were grown to mid-log phase (OD_600_ between 0.8 and 2). After two washes with PBS to remove the medium, the bacterial pellets were resuspended in sterile egg water, and the concentration was measured. Various bacterial concentrations were tested for their effect on survival. The final concentrations chosen were approximately 2.5 × 10⁸ CFU/ml for MAC 101 and 1.5 × 10⁸ CFU/ml for Mma20 which resulted in around 50 percent survival after 2.5 dpi. At 2.5 dpf (60 hpf), larvae from four experimental groups, GF *tlr2* wild-type group, GF *tlr2* mutant group, CONVD *tlr2* wild-type group and CONVD *tlr2* mutant group, were transferred to 96-well plates respectively, each well containing one larva. Bacteria were then added to each well and adjusted to the final concentration with a total volume of 0.3 ml per well. For MAC 101 immersion experiment, the larvae were incubated at 32 °C and for Mma20 immersion, the larvae were incubated at 28 °C. As MAC 101 has an optimal growth temperature closer to 37 ºC, whereas zebrafish larvae are typically maintained at 28 ºC. Incubation at 32 ºC is used as a compromise condition that maintains normal larval development while improving MAC 101 infection efficiency and reproducibility *in vivo*. Three biological duplications were conducted, with 20 larvae per group in each duplicate. At 2.5 days post-infection (dpi), corresponding to 5 dpf (120 hpf), all the survived larvae were transferred to egg water without bacteria for 4 hours to wash away the transiently colonized bacteria (GF larvae in sterile egg water, CONVD larvae in normal egg water). After washing, all larvae were imaged using the Leica Stellaris 5 confocal laser scanning microscope (Leica Microsystems, Wetzlar Germany).

To determine whether the MAC 101 and Mma20 bacteria infect zebrafish larvae through undamaged skin, we used agarose to block the head of larvae and then immersed the larvae with bacteria for 1 day. Supplementary Video 1 showed that the larva after immersion of the head in agarose was still alive. At 1 dpi (24 hpi), no bacteria were found inside the body of the head blocked larvae, showing that neither MAC 101 or Mma20 can infect zebrafish larvae through skin or via the cloaca (Supplementary Figure 1).

In addition, we observed that a small fraction of larvae displayed bacterial signal associated with the gill region after the natural immersion infection (Supplementary Figure 2). Prior to quantification, larvae showing detectable gill infection were excluded from downstream quantification and analysis.

**Figure 1:**
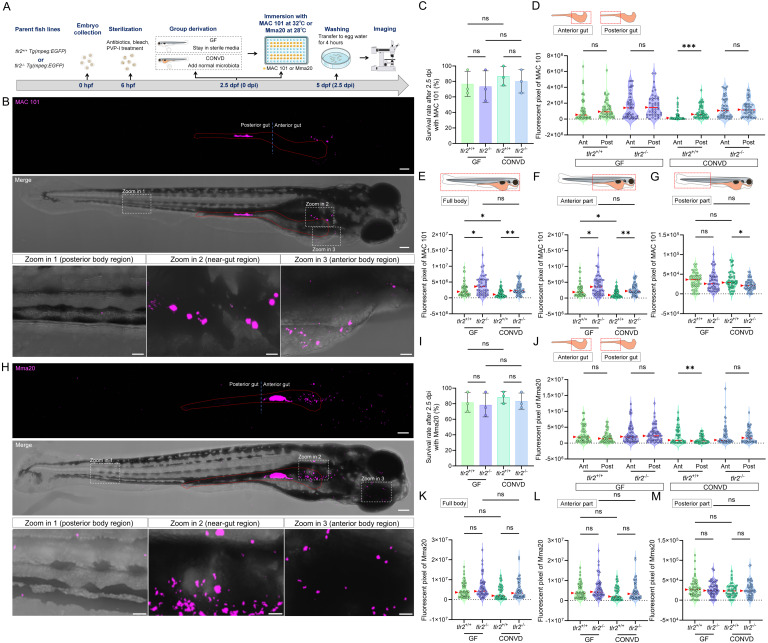
TLR2 and the microbiome modulate bacterial gut distribution and dissemination after immersion infection with MAC 101 and Mma20. (A) Schematic of the experimental workflow. Germ-free (GF) and conventionalized (CONVD) zebrafish larvae derived from *tlr2* wild-type and mutant lines were immersed in egg water containing either mCherry-labeled* M. avium complex* (MAC 101) or DsRed-labeled* M. marinum* (Mma20) bacteria for 2.5 days. (B) Representative fluorescence images showing MAC 101 localization primarily in the gut, with dissemination to anterior and posterior regions (representative images are *tlr2* wild-type larvae under the germ-free (GF) condition). Bacteria are shown in magenta. Scale bar: 100 µm for full-body images; 25 µm for zooms. (C) Survival rates for all groups after MAC 101 infection showing no significant differences between genotypes or microbial conditions. The results are based on 3 independent experiments. Error bars represent mean ± SD. (D) Distribution of MAC 101 within the gut shows preferential localization to the posterior gut in wild-type CONVD larvae, whereas this regional bias is lost in *tlr2* mutants and GF conditions. (E-F) Quantification of full body (E) and anterior region (F) bacterial burden (fluorescent pixel count) reveals significantly higher MAC 101 load in *tlr2* mutants compared to the wild type under both GF and CONVD conditions. (G) In the posterior body region, significantly more MAC 101 was detected in *tlr2* wild-type larvae than in mutants under CONVD conditions, but not under GF conditions. For MAC 101 infection, the data from GF *tlr2*^+/+^ (n=46) group, GF *tlr2*^-/-^ (n=44) group, CONVD *tlr2*^+/+^ (n=52) group and CONVD *tlr2*^-/-^ (n=48) group are based on three independent experiments. (H) Representative fluorescence images showing Mma20 localization primarily in the gut, with dissemination to anterior and posterior regions. (I) Survival rates for all groups after Mma20 infection showing no significant differences between genotypes or microbial conditions. The results are based on 3 independent experiments. Error bars represent mean ± SD. (J) Distribution of Mma20 in the gut shows higher abundance in the anterior gut in wild-type CONVD larvae, opposite to the distribution pattern observed with MAC 101. (K-M) Quantification of Mma20 bacterial burden in the full body (K) and anterior region (L) showing a non-significant trend toward higher levels in *tlr2* mutant larvae. (M) No significant differences in Mma20 burden were detected in the posterior region among groups. For Mma20 infection, the data from GF *tlr2*^+/+^ (n=50) group, GF *tlr2*^-/-^ (n=51) group, CONVD *tlr2*^+/+^ (n=45) group and CONVD *tlr2*^-/-^ (n=50) group are based on three independent experiments. Statistical significant difference was determined by one-way ANOVA, red arrows point to the median, ns, non-significant, *, *P* < 0.05, **, *P* < 0.01, ***, *P* < 0.001.

To study the bacterial distribution in the gut of MAC 101 and Mma20, we performed co-infection experiments with mWasabi-labeled MAC 101 and DsRed-labeled Mma20 for Casper fish line 50. At 2.5 dpf (60 hpf), larvae from GF or CONVD condition were immersed together with 0.5 × 10⁸ CFU/ml MAC 101 and 0.5 × 10⁸ CFU/ml Mma20 at 28 °C. After 2.5 dpi, all the survived larvae were transferred to egg water without bacteria for 4 hours to wash away the transiently colonized bacteria and then were imaged using the Leica Stellaris 5 confocal laser scanning microscope (Leica Microsystems, Wetzlar Germany). For each confocal image, the gut region of interest (ROI) was manually defined in ImageJ based on the anatomical boundaries of intestinal tissue visible in the image, as illustrated by the red dashed outlines in Figure 1B and 1H. Bacterial burden was quantified as the integrated fluorescence intensity in the fluorescent channel within each ROI. For spatial analysis of the gut, the gut ROI was operationally divided into anterior and posterior regions using consistent anatomical landmarks visible in larvae. The boundary was defined at the transition between the intestinal bulb and the straight intestinal segment, which is typically located near the posterior end of the swim bladder. The intestinal bulb anterior to this transition was designated as the anterior gut, and the intestinal tract posterior to this point was designated as the posterior gut (Figure 1B).

### 2.5. Robotic gut microinjection

Using the automated microinjection system for zebrafish larvae from Life Science Methods (Leiden, the Netherlands), we performed robotic bacterial injection to the gut lumen of larvae from four experimental groups (GF *tlr2* wild-type group, GF *tlr2* mutant group, CONVD *tlr2* wild-type group and CONVD *tlr2* mutant group) 51. The infection inoculum of bacteria was prepared in sterile PBS with 2% polyvinylpyrrolidone (PVP) 40 solution (Calbiochem, the Netherlands). PVP40 was included as an inert viscosity enhancer to stabilize the bacterial suspension, reduce settling, and improve injection reproducibility 15. Approximately 1000 colony-forming units (CFU) of MAC 101 bacteria and 250 CFU Mma20 bacteria were injected into the anterior bulb of the gut lumen at 4 dpf (96 hpf) for each larva. Given the pronounced developmental changes during this window, we strictly selected larvae at the same developmental stage and with a clearly formed intestinal bulb suitable for injection. The needle was inserted transmurally and the injection volume was 1 nl. To ensure that the extra-intestinal disseminations are due to natural invasion from the gut, but not due to the transmural injection process, we injected small volumes (1 nl) of a dyed inoculum (phenol red) into the gut lumen with a fine-tipped glass capillary needle. We only included larvae in which the bolus was clearly retained intraluminally without reflux along the needle tract. Any larvae showing dye leakage outside the gut were excluded before experiments. For all robotic gut microinjection in this study, the site of puncture is at the anterior bulb of the gut lumen as shown in Figure 3B. The gut injection process is shown in Supplementary Video 2. At 0.5 hpi, 2.5 hpi and 24 hpi, pools of injected larvae were collected and imaged using the Leica Stellaris 5 confocal laser scanning microscope (Leica Microsystems, Wetzlar Germany).

### 2.6. qRT-PCR detecting

We performed qRT-PCR on a CFX96TM Touch Real-Time PCR Detection (Bio-Rad Laboratories, Inc, USA) to detect the expression profiles of downstream immune related gene of TLR2 signaling for the larvae after 2.5 days immersion of MAC 101 and Mma20 and non-infected larvae as control. The peptidylprolyl isomerase A-like (*ppial*) gene was used as reference gene. The sequences of all the primers used in this study are showed in Supplementary Table 1. The qRT-PCR reaction procedure was performed using the following protocol: 95 °C 3 min, 40 cycles real time of 95 °C 15 sec, 68 °C 30 sec and 72 °C 30 sec, and final melting curve of 95 °C 1 min and 55 °C 10 sec. The qRT-PCR assay was performed in three independent biological replicates, and relative expression levels were calculated using the comparative 2^-ΔΔCt^ method 52.

### 2.7. Macrophage ablation using Metronidazole

For macrophage ablation experiments using Metronidazole (MTZ, Sigma-Aldrich), *Tg* (*mpeg:Gal4^gl25^; UAS:NTR-mCherry^c264^*) fish line (further refer to *mpeg:NTR* fish line) was used as macrophage ablated group. Prior to the start of the experiment, only *mpeg:NTR* larvae expressing strong fluorescence were screened for use in the experiment. *tlr2^+/+^ Tg* (*mpeg1*:* EGFP*)*^gl22^* fish line was used as control group. At 1 dpf (24 hpf), larvae from *mpeg:NTR* and *tlr2^+/+^* fish lines were treated with 5 mM MTZ by adding fresh MTZ to the egg water and then maintained in the dark as MTZ is sensitive to long light exposure 47. At 2.5 dpf (60 hpf), larvae from the *mpeg:NTR* group were screened using the Leica MZ16FA fluorescence stereo microscope (Leica Microsystems, Wetzlar Germany) for selecting the larvae without mCherry fluorescent signals. The absence of mCherry fluorescence signal indicates successful ablation of macrophages by MTZ. The larvae with macrophage ablation and the *tlr2^+/+^* larvae were then immersed with MAC 101 or Mma20 bacteria in 96-well plates following the same infection method as described previously. Two biological duplications were conducted.

To exclude a direct antibacterial effect of low-dose MTZ on MAC 101 or Mma20, we quantified bacterial burden in infected larvae treated with 5 mM MTZ and in untreated controls.

### 2.8. TLR2 inhibitor treatment

TLR2 antagonist C29 was purchased from MedChemExpress LLC (Herndon, VA, USA) and used in the TLR2 inhibition experiments 53. At 1 dpf (24 hpf), larvae from *tlr2^+/+^* fish line were treated with 1 µM or 5 µM C29 by adding the compound into the egg water. Larvae without any treatment served as control group. At 2.5 dpf (60 hpf), both C29 treated larvae and control larvae underwent the same immersion infection procedure, with the only difference being that the egg water for the C29 treated group continued to contain C29. Two biological duplications were conducted.

### 2.9. Confocal imaging and quantification

For the natural immersion infection, all survived larvae were collected and imaged using the Leica Stellaris 5 confocal laser scanning microscope (Leica Microsystems, Wetzlar Germany). The bacterial burden of the full body, anterior and posterior part of the body for the larvae from four experimental groups was quantified using ImageJ software. The anterior and posterior parts of the body were defined using the end of the gut as the anatomical boundary. The bacterial burden of the anterior and posterior half part of the gut was also quantified. The number of macrophages containing bacteria in the full body, anterior and posterior part of the body for the larvae was manually quantified. For the robotic gut injection, at 0.5 hpi, 2.5 hpi and 1 dpi, pools of injected larvae were collected and imaged using the Leica Stellaris 5 confocal laser scanning microscope. The bacterial burden and the macrophage number at the gut region were quantified using ImageJ software.

### 2.10. Live imaging and cell tracking

Time lapse imaging of 4 dpf (96 hpf)gut injected zebrafish larvae were visualized using the Leica Stellaris 5 confocal laser scanning microscope (Leica Microsystems, Wetzlar Germany) with 55 s time interval for 2 h using a 10× objective (N.A.0.75). PBS containing 2% PVP40 was injected the same volume as the bacteria as a control. The quantification for macrophage behavior was conducted in TrackMate of ImageJ. Using TrackMate, data of track displacement and mean speed of macrophages after gut injection were obtained. Track displacement represents the distance between the last spot of the track and the first spot of the track in time. Mean speed is defined as the track total distance traveled divided by the track total time.

### 2.11. Statistical analysis

The statistical analysis was performed using the Graphpad Prism software (Version 10; GraphPad Software, San Diego, CA, USA). D'Agostino-Pearson and Shapiro-Wilk normality test was performed to determine the Gaussian distribution of the data. All experimental data in this study are shown as mean ± the standard deviation (SD). The statistically significant differences between groups were determined using one-way analysis of variance (ANOVA) and the Tukey-Kramer method was used for post hoc analysis. The significance was established as * *P* < 0.05, ** *P* < 0.01, *** *P* < 0.001 and **** *P* < 0.0001.

## 3. Results

### 3.1. TLR2 and the microbiome modulate gut localization and dissemination of MAC 101

To investigate the function of TLR2 and the microbiome in host responses to NTM in the gut, we generated germ-free (GF) and conventionalized (CONVD) larvae from *tlr2* wild-type and mutant zebrafish lines and then immersed them under natural conditions with two NTM strains, *M. avium* strain MAC 101 and *M. marinum* strain Mma20 (Figure 1A). In the absence of infection, neither *tlr2* genotype nor microbiome status affected larval survival, and uninfected larvae showed no mortality over the same time window. With the chosen bacterial concentrations, no significant differences in survival rate were observed between *tlr2* mutant and wild-type larvae in either GF or CONVD condition after infection with either bacterial strain (Figure 1C,I). After 2.5 days of immersion infection with the both strains, bacteria were predominantly localized in the gut area, but were also detectable outside the gut in both the anterior and posterior body regions (Figure 1B,H). When the head was immersed in agarose, there was no spread of bacteria inside the body, showing an absolute dependency of oral uptake for the spread of MAC 101 or Mma20 inside the body (Supplementary Figure 1).

Quantitative measurement of the distribution of *M. avium* strain MAC 101 bacteria in the gut showed that for the wild-type microbiome-colonized larvae, the bacteria were present in higher quantities in the posterior part of gut than in the anterior part. However, in the *tlr2* mutant larvae or under germ free conditions, there was no significant difference in the distribution of bacteria in the anterior and posterior parts of the gut (Figure 1D). This shows that both TLR2 and the microbiome influence spatial bacterial distribution of MAC 101 bacteria in the gut.

Since MAC 101 bacteria also disseminated outside the gut, we also performed further quantification of bacterial fluorescence in the entire body. The results showed that bacteria were only extremely rarely found inside the gill area (Supplementary Figure 2) indicating that bacteria outside the gut where predominantly coming from the gut lumen. Given the limitation of confocal imaging in resolving the exact anatomical localization of extra-lumenal bacterial signal, we did not assign bacteria to specific tissues within the body. Instead, we quantified spatial distribution at the body-region level by partitioning the larva into anterior and posterior regions. The analysis of the total and anterior part of the body showed a significantly higher bacterial burden in the *tlr2* mutant larvae compared to the wild type under both GF and CONVD conditions (Figure 1E,F). In the posterior part, more MAC 101 bacteria were detected in *tlr2* wild-type larvae compared to the mutant under the microbiome-colonized condition, but this was not significant in GF larvae (Figure 1G). These results show that bacteria can disseminate out of the gut into the body which is partially dependent on *tlr2* and that the microbiome influences distal dissemination. In wild-type larvae, the GF condition showed a higher bacterial burden in the full body and the anterior part compared to CONVD larvae, demonstrating the protective function of the microbiome against infection by MAC 101 (Figure 1E,F). However, this protective role was not observed in the *tlr2* mutant larvae (Figure 1E,F).

**Figure 2:**
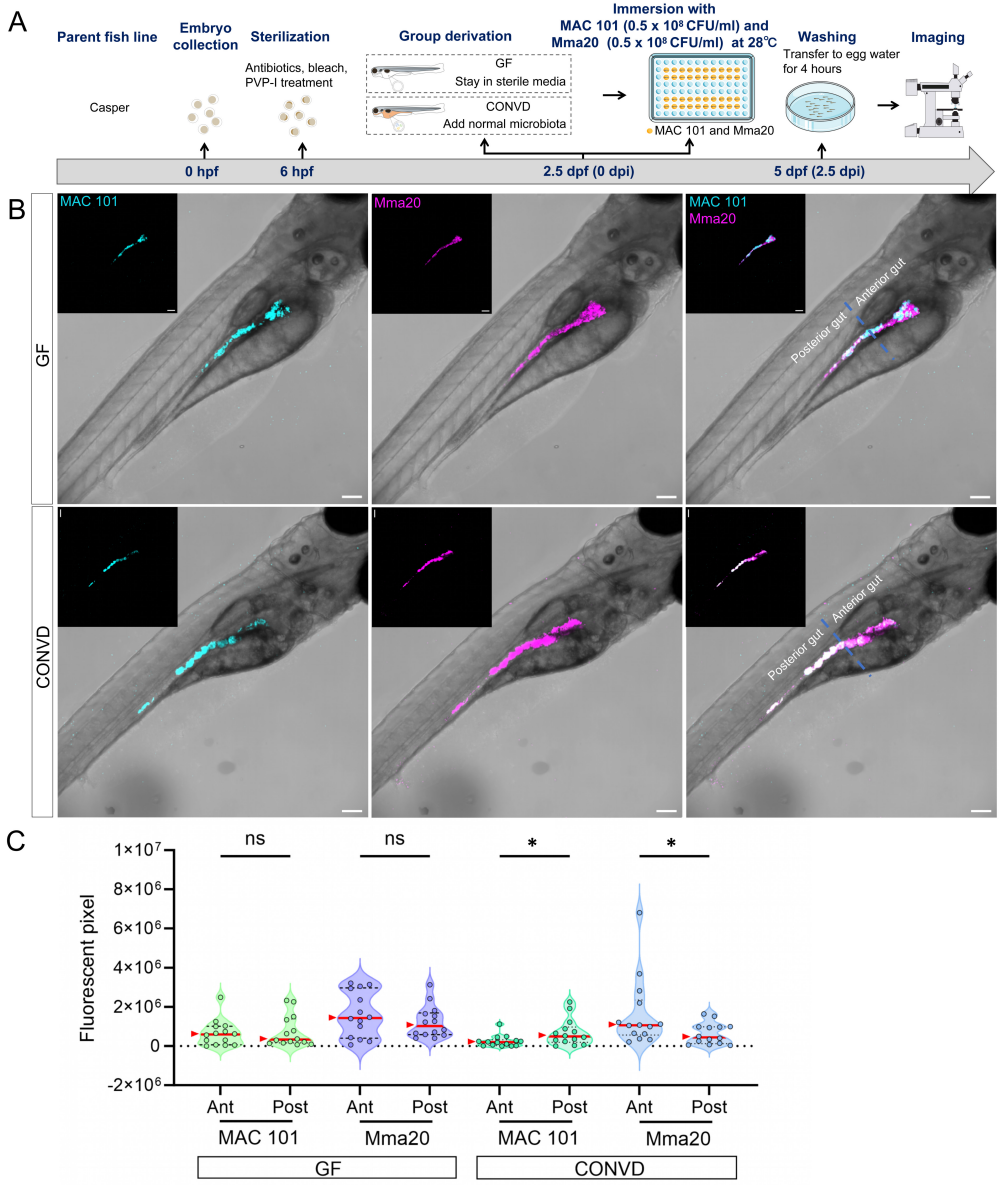
Co-infection experiments show distinct spatial colonization patterns of MAC 101 and Mma20 bacteria. (A) Schematic of the experimental workflow. Germ-free (GF) and conventionalized (CONVD) zebrafish larvae derived from Casper fish line were immersed in egg water containing mWasabi-labeled* M. avium* (MAC 101) and DsRed -labeled *M. marinum* (Mma20) at the same time for 2.5 days. (B) Representative fluorescence images show that MAC 101 preferentially localizes to the posterior part of gut and Mma20 preferentially localizes to the anterior part of gut in CONVD larvae, whereas this regional bias is lost in GF conditions. MAC 101 bacteria are shown in cyan; Mma20 bacteria are shown in magenta. Scale bar: 100 µm. The data from GF (n=14) group and CONVD (n=14) group are based on two independent experiments. Statistical significant difference was determined by one-way ANOVA, red arrows point to the median, ns, non-significant, *, *P* < 0.05, **, *P* < 0.01, ***, *P* < 0.001.

Interestingly, for the *M. marinum* strain Mma20 infection, analysis of the distribution of Mma20 bacteria showed that Mma20 was more abundant in the anterior gut than the posterior gut in wild-type microbiome-colonized larvae (Figure 1J). This regional bias differed from that observed for MAC 101, which was more enriched in the posterior gut in the same condition (Figure 1D). This result was corroborated by mixed inoculation of both MAC 101 and Mma20 strains expressing different fluorescent GFP varieties (Figure 2). However, in the *tlr2* mutant larvae or under germ free conditions, there was no significant difference in the distribution of bacteria in the anterior and posterior parts of the gut (Figure 1J). This shows that both TLR2 and the microbiome influence spatial bacterial distribution for both MAC 101 and Mma20 bacteria in the gut. This also implies that in the absence of *tlr2* or a microbiome there is no difference anymore in the gut microbial distribution between the two bacterial strains.

In contrast to the results with MAC 101, with Mma20 quantification of the fluorescent pixels of bacteria in the full body, the anterior part and posterior part, showed no significant differences between wild type and *tlr2* mutant or GF and CONVD conditions, only with an elevated trend in GF larvae compared with CONVD larvae in wild type (Figure 1K-M). In conclusion, the protection against proliferation and dissemination of Mma20 after oral infection is not strictly dependent on* tlr2* or the microbiome, as is not the case with MAC 101.

### 3.2. Gut microinjection confirms the roles of TLR2 and the microbiome in MAC 101 and Mma20 proliferation

**Figure 3:**
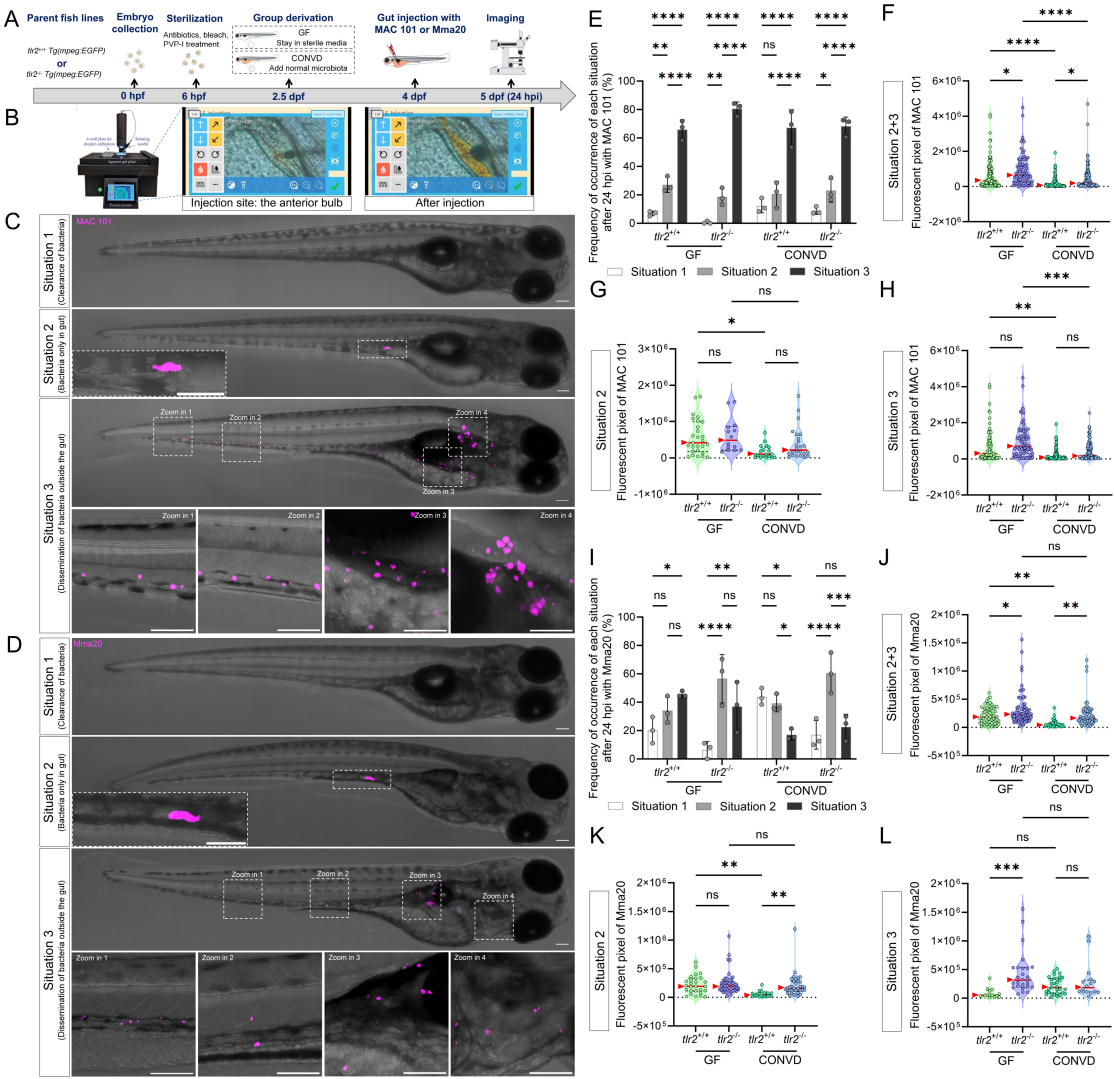
Gut microinjection confirms the roles TLR2 and the microbiome in MAC 101 and Mma20 proliferation. (A) Schematic of the gut microinjection procedure. (B) Robotic gut microinjection. The injection site is the area of the anterior bulb of the gut. Successful injection was characterized by the absence of extra-intestinal leakage of bacteria. (C,D) Representative fluorescence images showing three situations after 24 hours of gut microinjection of MAC 101 (C) and Mma20 (D). Representative images are *tlr2* wild-type larvae under germ-free (GF) condition. Bacteria are shown in magenta. Situation 1 represents clearance of bacteria, Situation 2 represents retention of bacteria within the gut, and Situation 3 represents dissemination of bacteria outside the gut. Scale bar: 100 µm for full-body images; 25 µm for zooms. (E,I) Frequency of each situation after 24 hours of gut microinjection of MAC 101 (E) and Mma20 (I). (F,J) Total bacterial burden calculated by combining situations 2 and 3 for MAC 101 (F) and Mma20 (J). (G,K) Bacterial burden of situations 2 for MAC 101 (G) and Mma20 (K). (H,L) Bacterial burden of situations 3 for MAC 101 (H) and Mma20 (L). For MAC 101 infection, the data from the GF *tlr2*^+/+^ (n=103) group, GF *tlr2*^-/-^ (n=79) group, CONVD *tlr2*^+/+^ (n=92) group and CONVD *tlr2*^-/-^ (n=110) group are based on three independent experiments. For Mma20 infection, the data from GF *tlr2*^+/+^ (n=79) group, GF *tlr2*^-/-^ (n=72) group, CONVD *tlr2*^+/+^ (n=59) group and CONVD *tlr2*^-/-^ (n=62) group are based on three independent experiments. Statistical significant difference was determined by one-way ANOVA, red arrows point to the median, ns, non-significant, *, *P* < 0.05, **, *P* < 0.01, ***, *P* < 0.001, ****, *P* < 0.0001.

To further validate the findings from immersion-based natural infection, we employed a novel robotic gut microinjection approach to directly introduce NTM into the intestinal tract with a controllable dose of bacteria (Figure 3A,B). Since MAC 101 and Mma20 display different replication kinetics in zebrafish larvae, we used inocula of 1000 CFU (MAC101) and 250 CFU (Mma20), which were empirically chosen to yield robust, reproducible infections that elicit host immune responses 15. 24 hours after *M. avium* strain MAC 101 gut microinjection, three ranges of situations could be classified: situation 1: complete clearance of bacteria from the gut; situation 2: retention of bacteria within the gut and situation 3: dissemination of bacteria to tissues outside the gut (Figure 3C). Most larvae from all four experimental groups exhibited situation 3, with very few larvae in situation 1 in any condition (Figure 3E). When combining the pixel counts for all larvae with situation 2 and 3, *tlr2* mutant larvae exhibited a higher bacterial burden compared to wild-type larvae under both GF and CONVD conditions. Furthermore, GF larvae had a higher bacterial burden compared to CONVD larvae in both *tlr2* wild-type and mutant larvae (Figure 3F). When comparing the pixel counts of larvae exhibited situation 2, GF larvae showed a higher bacterial burden compared to CONVD larvae in *tlr2* wild-type larvae (Figure 3G). When comparing the pixel counts of larvae exhibited situation 3, GF larvae had a higher bacterial burden compared to CONVD larvae in both *tlr2* wild-type and mutant larvae (Figure 3H).

Similarly, after 24 hours of *M. marinum* strain Mma20 gut microinjection, the same 3 situations were found (Figure 3D, I). When comparing the pixel counts of larvae exhibited situation 2, GF larvae showed a higher bacterial burden compared to CONVD larvae in *tlr2* wild-type larvae. *tlr2* mutant larvae exhibited a higher bacterial burden compared to wild-type larvae under CONVD conditions (Figure 3K). When comparing the pixel counts of larvae exhibited situation 3, *tlr2* mutant larvae exhibited a higher bacterial burden compared to wild-type larvae under CONVD conditions (Figure 3L). Consistent with MAC 101, in the combined data of pixel counts from situation 2 and 3, *tlr2* mutant larvae had higher bacterial burden compared to wild-type larvae under both GF and CONVD conditions. As in the MAC 101 study, GF larvae had higher bacterial burden compared to CONVD larvae in *tlr2* wild-type larvae, while there was no significant difference in mutant larvae (Figure 3J). These results show that TLR2 and the microbiome contribute to host suppression of NTM colonization in infection by MAC 101 and Mma20. These results with MAC 101 are confirming the data found with the oral infection method. However, with Mma20 we did not detect an effect of TLR2 mutation or the microbiome after oral dosing of the bacteria. Apparently, gut injection provides a higher discriminating power to observe bacterial proliferation characteristics in the case of Mma20 infection.

### 3.3. TLR2 and the microbiome modulate innate immune gene expression during MAC 101 and Mma20 infection

**Figure 4:**
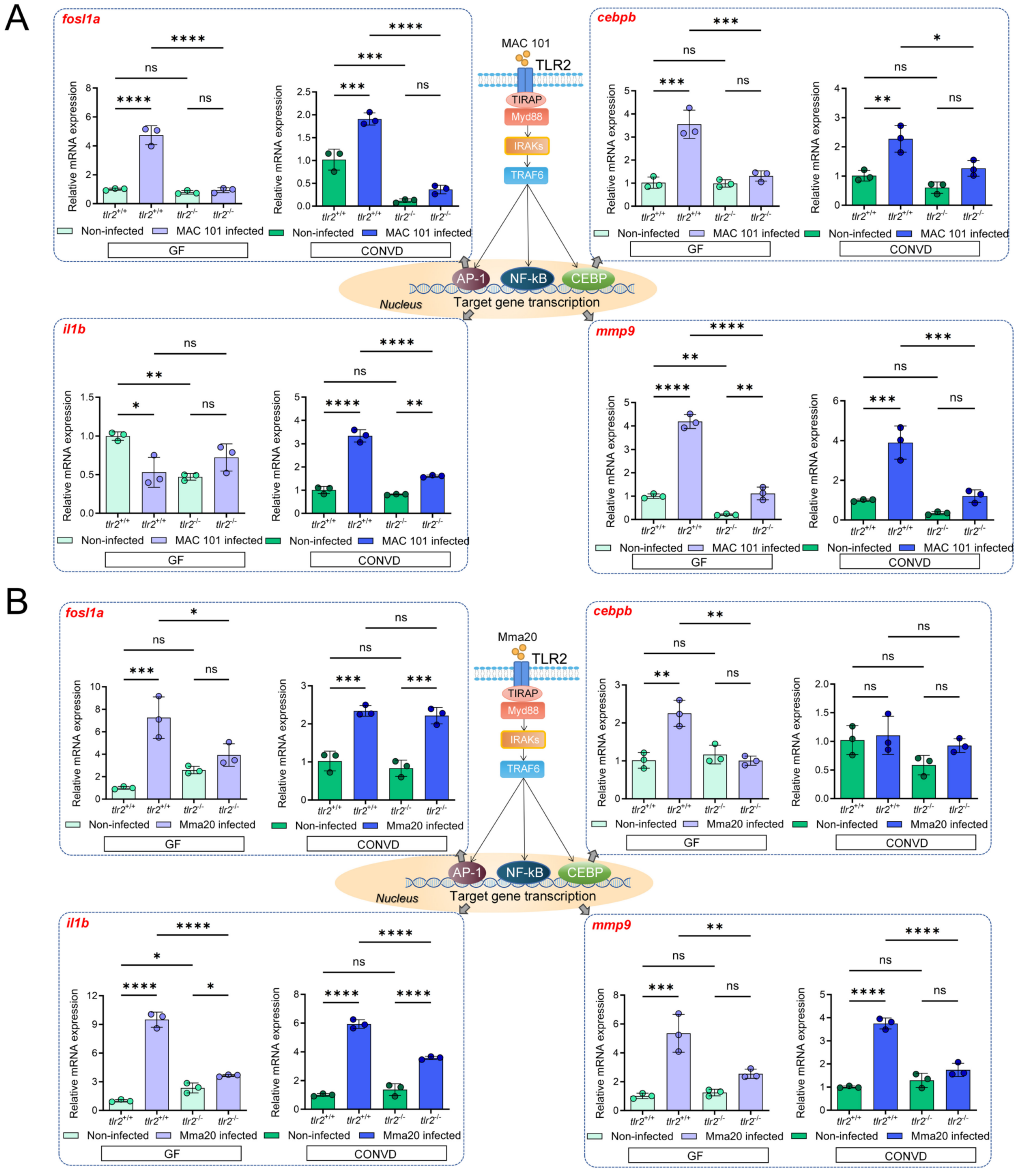
Immune-related gene expression profiles after MAC 101 and Mma20 immersion infection. (A) Immune-related gene expression profiles after MAC 101 immersion infection. (B) Immune-related gene expression profiles after Mma20 immersion infection. Statistical significant difference was determined by one-way ANOVA, ns, non-significant, *, *P* < 0.05, **, *P* < 0.01, ***, *P* < 0.001, ****, *P* < 0.0001.

To further investigate the molecular mechanisms by which TLR2 and the microbiome influence host responses, the relative expression of several downstream genes associated with TLR2 signaling was examined in zebrafish larvae after MAC 101 and Mma20 immersion infection. These genes included FOS-like antigen 1a (*fosl1a*), CCAAT/enhancer-binding protein beta (*cebpb*), interleukin 1 beta (*il1b*) and matrix metallopeptidase 9 (*mmp9*), all known to be involved in innate immune responses to mycobacterial infection in zebrafish larvae 31, 54, 55. In Figure 4, we presented the *tlr2* genotype comparison (*tlr2* mutant versus wild type) within each microbiome condition (GF and CONVD). Supplementary Figure 3 reformatted the same dataset to emphasize the microbiome comparison (CONVD versus GF) within each genotype (*tlr2* wild type and mutant). We first performed qRT-PCR analysis on larvae after MAC 101 immersion infection. For *fosl1a*, *cebpb* and *mmp9*, the relative gene expression in *tlr2* wild-type larvae was significantly up-regulated after 2.5 days of MAC 101 immersion infection under both GF and CONVD conditions. However, in the *tlr2* mutant after infection there was no significant upregulation in either GF or CONVD condition for *fosl1a* and *cebpb* (Figure 4A). For *mmp9* in *tlr2* mutant larvae there was a low but significant upregulation under the GF condition but not in the CONVD condition after infection. This suggests that the response of *fosl1a*, *cebpb* and *mmp9* as downstream genes after MAC 101 immersion infection is dependent on TLR2 and this is also influenced by the microbiome (Supplementary Figure 3). For *il1b*, the *tlr2* wild-type larvae had an upregulated relative gene expression after infection under the CONVD condition, yet showed an unexpected down regulation under the GF condition. This downregulation of *Il1b* after infection under the GF condition was not observed in the *tlr2* mutant (Figure 4A).

We then performed qRT-PCR on larvae after Mma20 immersion infection. For *fosl1a*, *cebpb, il1b* and *mmp9*, the relative gene expression in *tlr2* wild-type larvae was upregulated after 2.5 days of Mma20 immersion infection under both GF and CONVD conditions, except for *cebpb* in the CONVD condition. However, in the *tlr2* mutant there was no significant upregulation in either GF or CONVD condition for* mmp9* and* cebpb* (Figure 4B). For *fosl1a*, there was still significant upregulation in the *tlr2* mutant in the CONVD condition, but not in the GF condition. The *il1b* gene was still upregulated after infection in both GF and CONVD conditions (Figure 4B). This suggests that the response of TLR2 downstream genes after Mma20 immersion infection is dependent on TLR2 and on the microbiome. These findings highlight distinct patterns of TLR2-dependent gene regulation after gut infection by MAC 101 and Mma20 and suggest that the microbiome context in both cases influences specific transcriptional responses.

### 3.4. Macrophages are a key factor in the dissemination of MAC 101 and Mma20 bacteria to the posterior body region

**Figure 5:**
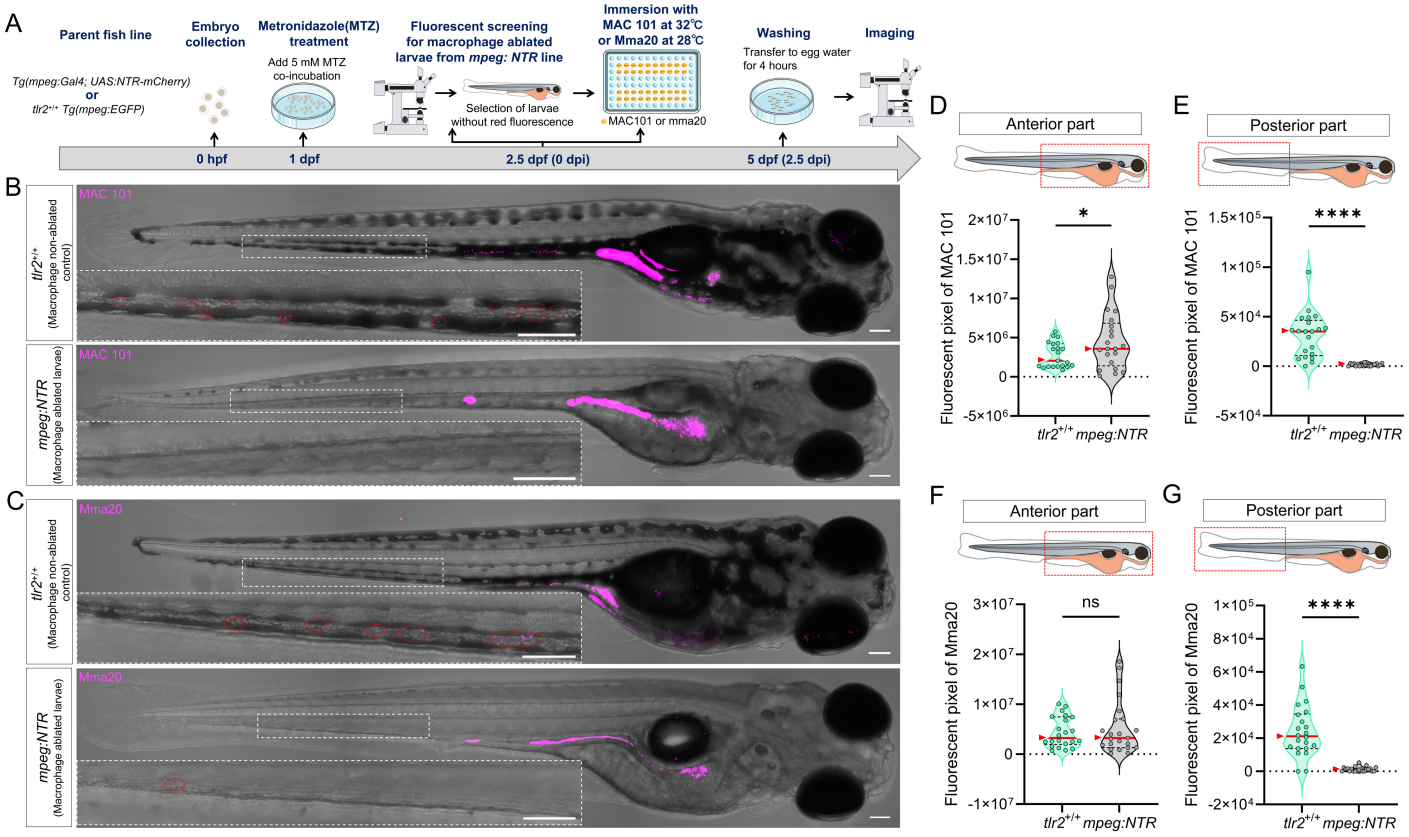
Macrophages are a key factor in the dissemination of MAC 101 and Mma20 bacteria to posterior body region. (A) Schematic of the experimental workflow. Zebrafish larvae were treated with 5 mM metronidazole (MTZ) at 1 dpf. After screening for the successful macrophage-ablated larvae, they were immersed with mCherry-labeled MAC 101 or DsRed-labeled Mma20 at 2.5 dpf for 2.5 dpi. (B,C) Representative fluorescence images showing bacterial dissemination in *tlr2* wild-type and macrophage-ablated larvae after infection with MAC 101 (B) and Mma20 (C). Bacteria are shown in magenta. Scale bar: 100 µm. (D,E) Quantification of the bacterial burden in the anterior region (D) and the posterior region (E) after infection with MAC 101. (F,G) Quantification of the bacterial burden in the anterior region (F) and the posterior region (G) after infection with Mma20. Macrophage-ablated larvae exhibited significantly fewer bacteria in the posterior part of the body compared to non-ablated control larvae. For MAC 101 infection, the data from the *tlr2*^+/+^ wild-type (n=21) group and macrophage-ablated group (n=21) are based on two independent experiments. For Mma20 infection, the data from the *tlr2*^+/+^ wild-type (n=22) group and macrophage-ablated group (n=23) are also based on two independent experiments. Statistical significant difference was determined by one-way ANOVA, red arrows point to the median, ns, non-significant, *, *P* < 0.05, ****, *P* < 0.0001.

To investigate the role of macrophages in MAC 101 and Mma20 bacterial dissemination from the gut, we compared bacterial distribution in normal and macrophage-ablated zebrafish larvae after MAC 101 and Mma20 immersion infection (Figure 5A-C). For this, metronidazole (MTZ) was used to treat the *Tg* (*mpeg:Gal4^gl25^; UAS:NTR-mCherry^c264^*) fish line for macrophage ablation 47. The *tlr2^+/+^ Tg* (*mpeg*:* EGFP*)*^gl22^* line was used as control group to test the effect of MTZ on bacterial survival. The results showed that 5 mM MTZ treatment did not significantly alter the bacterial burden for either MAC 101 or Mma20 after immersion infection, indicating that 5 mM MTZ does not measurably inhibit these mycobacteria *in vivo* in our assay (Supplementary Figure 4).

Quantification revealed region-dependent effects of macrophage ablation. For the anterior part, macrophage-ablated larvae showed a higher bacterial burden compared to the controls after MAC 101 immersion infection (Figure 5D), whereas there was no significant difference after Mma20 immersion infection (Figure 5F). However, bacterial signal in the posterior body region was markedly reduced in macrophage-ablated larvae relative to controls after both MAC 101 and Mma20 immersion infection (Figure 5E,G). These results show that macrophages are not only key to immune recognition and phagocytosis, but also contribute to the dissemination of bacteria from the gut to posterior body region.

### 3.5. TLR2 regulates macrophage-based MAC 101 and Mma20 dissemination via the gut

**Figure 6:**
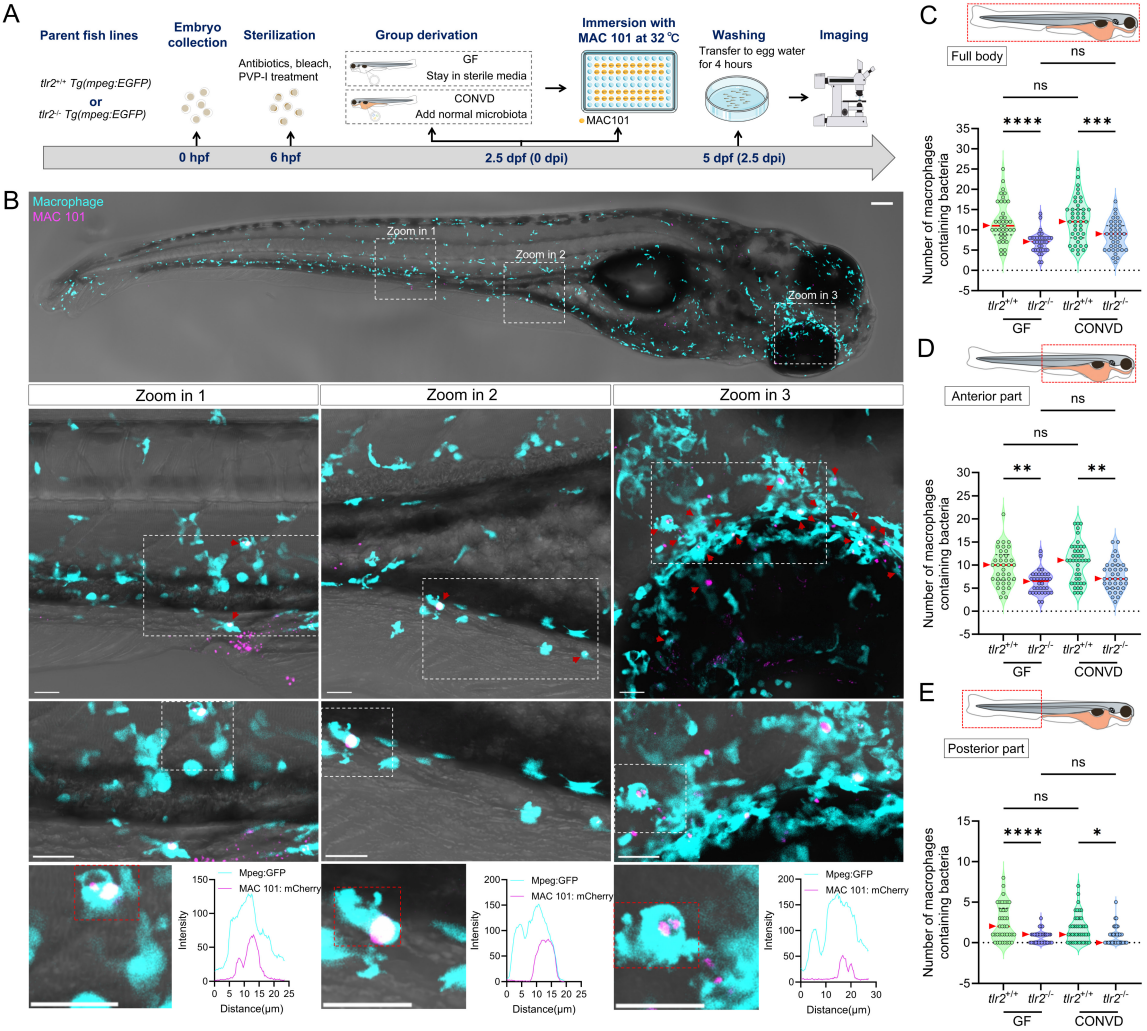
TLR2, but not the microbiome, determines macrophage load of MAC 101 from the gut in zebrafish larvae. (A) Schematic of the experimental workflow. (B) Representative fluorescence images showing macrophages containing MAC 101 bacteria in different body regions (representative images are *tlr2* wild-type larvae under germ-free (GF) condition). Bacteria are shown in magenta. Macrophages are shown in cyan. Scale bar: 100 µm for full-body images; 25 µm for zooms. The bottom graphs illustrate the spatial colocalization patterns of representative macrophages and bacteria. (C-E) Quantification of the number of bacteria-containing macrophages in the full body (C), anterior region (D), and posterior region (E). Wild-type larvae had significantly more infected macrophages than *tlr2* mutants under both GF and CONVD conditions in all regions. No significant differences were observed between GF and CONVD groups within the same genotype. Macrophages containing bacteria were manually counted across the full z-stack for each region. Each z-slice was examined to verify that individual bacteria-containing macrophages were counted once and only once. The data from GF *tlr2*^+/+^ (n=38) group, GF *tlr2*^-/-^ (n=34) group, CONVD *tlr2*^+/+^ (n=43) group and CONVD *tlr2*^-/-^ (n=37) group are based on three independent experiments. Statistical significant difference was determined by one-way ANOVA, red arrows point to the median, ns, non-significant, *, *P* <0.05, **, *P* < 0.01, ***, *P* < 0.001, ****, *P* < 0.0001.

**Figure 7:**
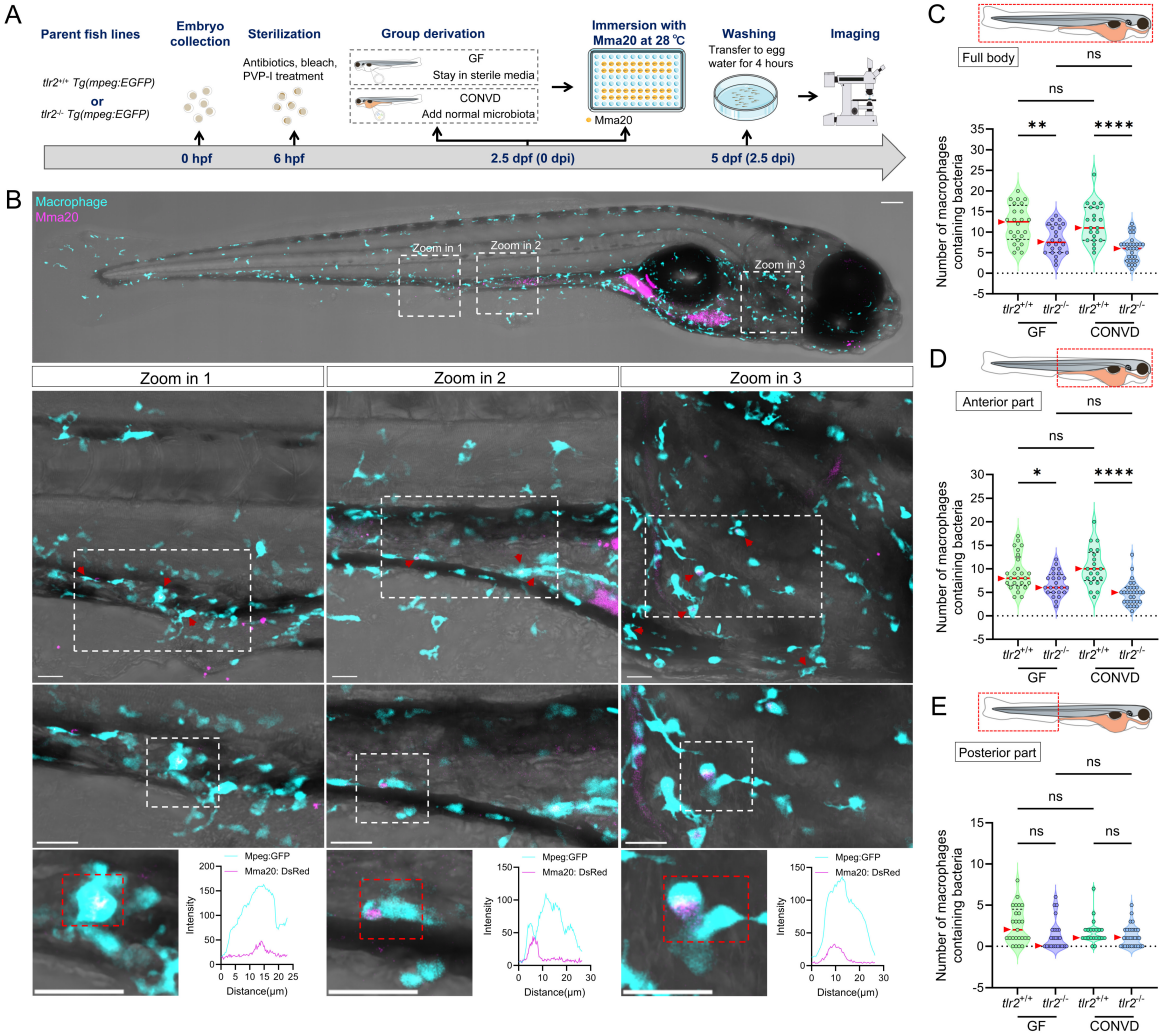
TLR2, but not the microbiome, determines macrophage load of Mma20 from the gut in zebrafish larvae. (A) Schematic of the experimental design. (B) Representative fluorescence images showing macrophages containing Mma20 bacteria in different body regions (representative images are *tlr2* wild-type larvae under germ-free (GF) condition). Bacteria are shown in magenta. Macrophages are shown in cyan. Scale bar: 100 µm for full-body images; 25 µm for zooms. The bottom graphs illustrate the spatial colocalization patterns of macrophages and bacteria. (C-E) Quantification of the number of bacteria-containing macrophages in the full body (C), anterior region (D), and posterior region (E). Wild-type larvae had significantly more infected macrophages than *tlr2* mutants under both GF and CONVD conditions in the full body and anterior regions, with no significant differences between GF and CONVD conditions. For the posterior body region, no significance was found among groups. Macrophages containing bacteria were manually counted across the full z-stack for each region. Each z-slice was examined to verify that individual bacteria-containing macrophages were counted once and only once. The data from GF *tlr2*^+/+^ (n=25) group, GF *tlr2*^-/-^ (n=21) group, CONVD *tlr2*^+/+^ (n=24) group and CONVD *tlr2*^-/-^ (n=30) group are based on two independent experiments. Statistical significant difference was determined by one-way ANOVA, red arrows point to the median, ns, non-significant, *, *P* < 0.05, **, *P* < 0.01, ****, *P* < 0.0001.

To further study whether TLR2 and the microbiome influence the response of immune cells upon MAC 101 and Mma20 infection, we quantified the number of macrophages that contained bacteria in different body regions, including full body, anterior and posterior part of the body (Figure 6A,B for MAC 101; Figure 7A,B for Mma20). To validate that bacterial fluorescence was contained within macrophages, we provided orthogonal (xz, yz) views in Supplementary Figure 5, Supplementary Video 3 further confirmed the co-localization of macrophage and bacterial signals. After MAC 101 immersion infection, more macrophages that contain bacteria were found in the *tlr2* wild-type larvae than in the mutants under both GF and CONVD conditions in all analyzed regions. There was no significant difference between the number of macrophages that contain bacteria in GF and CONVD larvae in either *tlr2* wild-type or mutant genotype (Figure 6C-E). Similarly, after Mma20 immersion infection, the number of infected macrophages in the full body and anterior regions was higher in *tlr2* wild-type larvae than in mutant larvae, with no significant differences between GF and CONVD conditions (Figure 7C,D). However, for the posterior body region, no significance was found among groups (Figure 7E). In conclusion, accumulation of MAC 101 and Mma20 bacteria in macrophages depends on *tlr2* which is not dependent on the microbiome. Distant dissemination of gut bacteria to the tail area is also dependent on* tlr2* in the case of MAC 101, but this could not be proven for Mma20.

**Figure 8:**
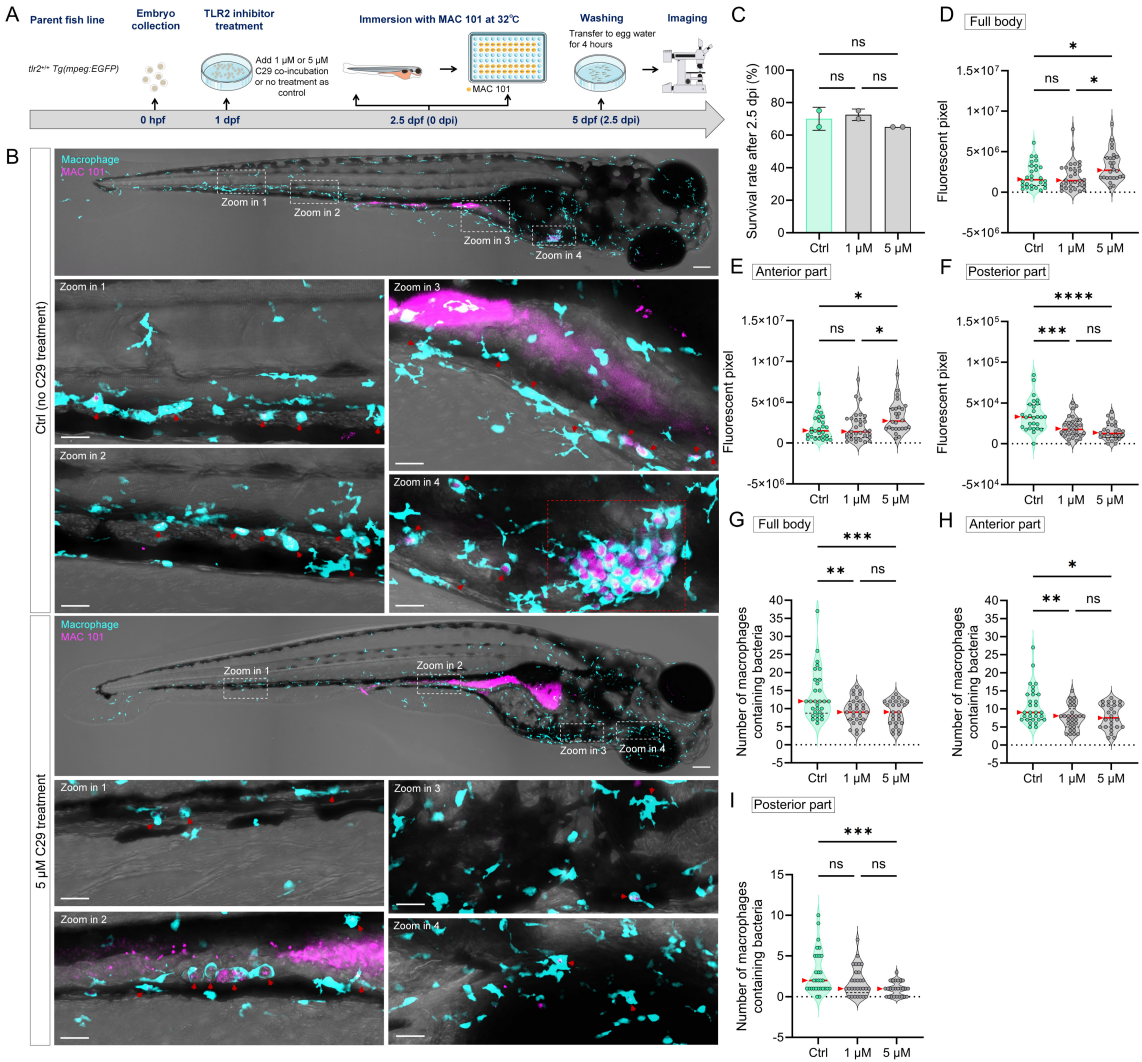
TLR2 chemical inhibition impairs macrophage-mediated dissemination of MAC 101 bacteria in zebrafish larvae. (A) Schematic of the experimental workflow. Zebrafish larvae were treated with the TLR2 inhibitor C29 at 1 dpf and then immersed with mCherry-labeled MAC 101 at 2.5 dpf for 2.5dpi. (B) Representative fluorescence images showing macrophages containing MAC 101 bacteria in different body regions in *tlr2* wild-type and C29 treated larvae. Bacteria are shown in magenta. Macrophages are shown in cyan. Red dashed box represents suspected granuloma structures. Scale bar: 100 µm for full-body images; 25 µm for zooms. (C) Survival rates for all groups after MAC 101 infection showing no significant differences between the wild type and C29 treated larvae. The results are based on 3 independent experiments. Error bars represent mean ± SD. (D, E) Quantification of full body (D) and anterior region (E) bacterial burden (fluorescent pixel count) reveals significantly higher MAC 101 load in 5 µM C29 treated larvae compared to the wild type and 1 µM C29 treated larvae. (F) In the posterior body region, significantly more MAC 101 was detected in tlr2 wild-type larvae than 1 µM or 5 µM C29 treated larvae. (G-I) Quantification of the number of bacteria-containing macrophages in the full body (G), anterior region (H), and posterior region (I). Wild-type larvae had significantly more infected macrophages than 1 µM or 5 µM C29 treated larvae in full body and anterior region. For posterior region, wild-type larvae have more infected macrophages than 5 µM C29 treated larvae. Macrophages containing bacteria were manually counted across the full z-stack for each region. Each z-slice was examined to verify that individual bacteria-containing macrophages were counted once and only once. The data from *tlr2*^+/+^ wild-type (n=27) group, 1 µM C29 treated (n=31) group and 5 µM C29 treated group (n=26) are based on two independent experiments. Statistical significant difference was determined by one-way ANOVA, red arrows point to the median, ns, non-significant, *, *P* < 0.05, **, *P* < 0.01, ***, *P* < 0.001, ****, *P* < 0.0001.

To further confirm the regulatory role of TLR2 in macrophage function during MAC 101 and Mma20 infection, we performed TLR2 inhibition experiments using the specific TLR2 antagonist C29 53. Wild-type zebrafish larvae were treated with C29 prior to immersion infection with either MAC 101 or Mma20 under conventionalized conditions, and with the used concentration there was no significant difference in survival between the groups after infection (Figure 8A-C). For MAC 101 infection, quantification of fluorescent bacterial signals revealed a significantly higher total and anterior bacterial burden in the C29-treated larvae compared to the wild type (Figure 8D,E). In the posterior part, more MAC 101 bacteria were detected in untreated larvae compared to the inhibitor-treated larvae (Figure 8F). Furthermore, inhibition of TLR2 led to a significant reduction in the number of macrophages containing MAC 101 bacteria. The results confirm the results observed in *tlr2* mutant larvae after immersion infection (Figure 8G-I).

**Figure 9:**
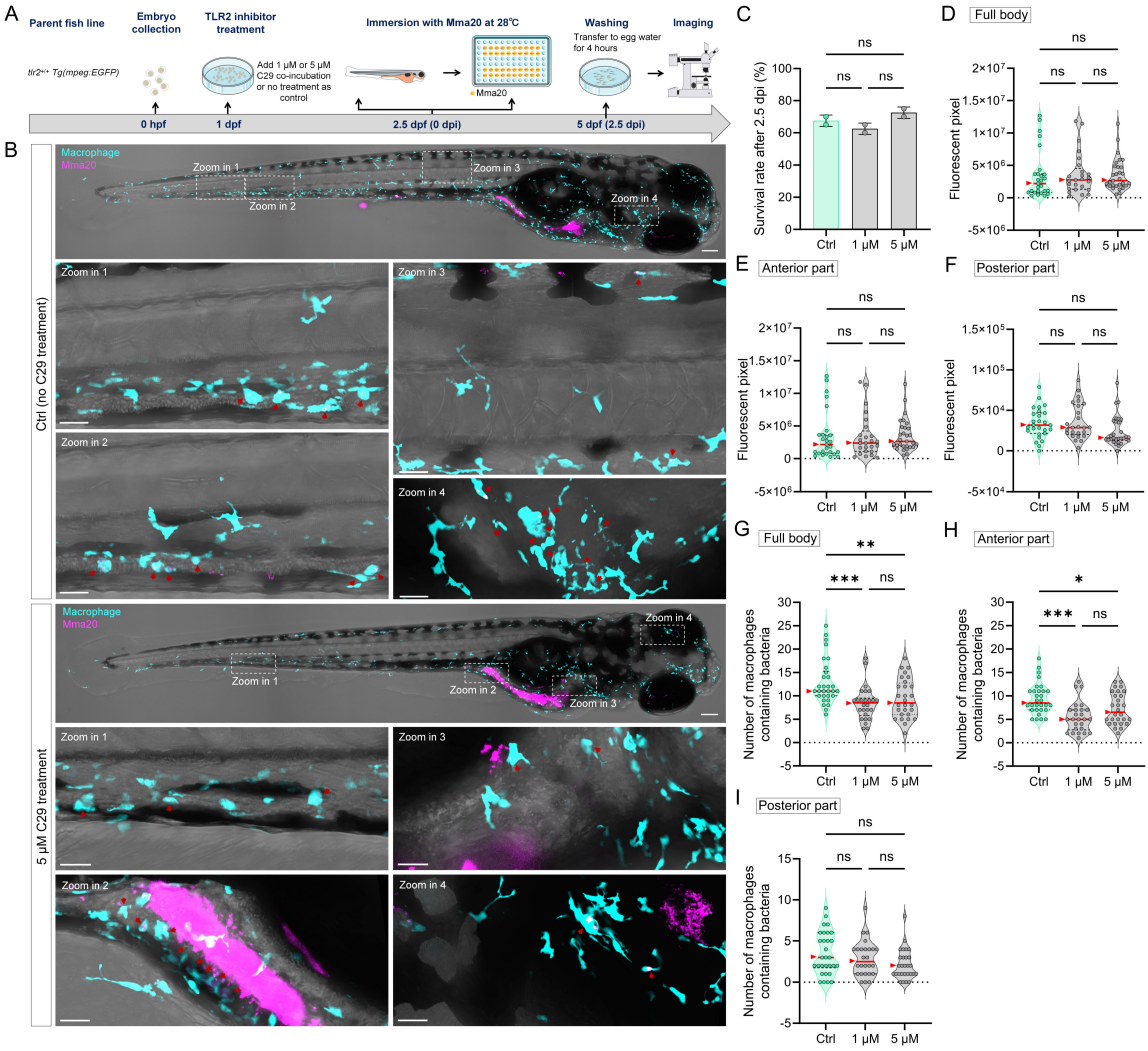
TLR2 chemical inhibition impairs macrophage-mediated dissemination of Mma20 bacteria in zebrafish larvae. (A) Schematic of the experimental workflow. Zebrafish larvae were treated with the TLR2 inhibitor C29 at 1 dpf and then immersed with DsRed-labeled Mma20 at 2.5 dpf for 2.5dpi. (B) Representative fluorescence images showing macrophages containing Mma20 bacteria in different body regions in *tlr2* wild-type and C29 treated larvae. Bacteria are shown in magenta. Macrophages are shown in cyan. Scale bar: 100 µm for full-body images; 25 µm for zooms. (C) Survival rates for all groups after Mma20 infection showing no significant differences between the wild type and C29 treated larvae. The results are based on 3 independent experiments. Error bars represent mean ± SD. (D-F) Quantification of full body (D), anterior region (E) and posterior region (F) bacterial burden (fluorescent pixel count) shows no significant difference between the wild type and 1 µM or 5 µM C29 treated larvae. (G-I) Quantification of the number of bacteria-containing macrophages in the full body (G), anterior region (H), and posterior region (I). Wild-type larvae had significantly more infected macrophages than 1 µM or 5 µM C29 treated larvae in full body and anterior region. For posterior region, there is no significant difference between wild-type larvae and 1 µM or 5 µM C29 treated larvae. Macrophages containing bacteria were manually counted across the full z-stack for each region. Each z-slice was examined to verify that individual bacteria-containing macrophages were counted once and only once. The data from *tlr2*^+/+^ wild-type (n=27) group, 1 µM C29 treated (n=25) group and 5 µM C29 treated group (n=29) are based on two independent experiments. Statistical significant difference was determined by one-way ANOVA, red arrows point to the median, ns, non-significant, *, *P* < 0.05, **, *P* < 0.01, ***, *P* < 0.001.

For Mma20 infection, the difference in bacterial burden between the groups was not significant in all analyzed regions (Figure 9A-F). The number of infected macrophages in the full body and anterior regions was higher in untreated larvae than in C29-treated larvae. However, for the posterior body region, no significance was found among the groups (Figure 9G-I). These results are also consistent with that of *tlr2* mutant larvae after immersion infection. These findings further confirm the regulatory role of TLR2 in mediating macrophage phagocytosis of MAC 101 and Mma20 bacteria after gut infection.

### 3.6. TLR2 deficiency hampers early macrophage accumulation at the gut after MAC 101 and Mma20 gut microinjection

**Figure 10:**
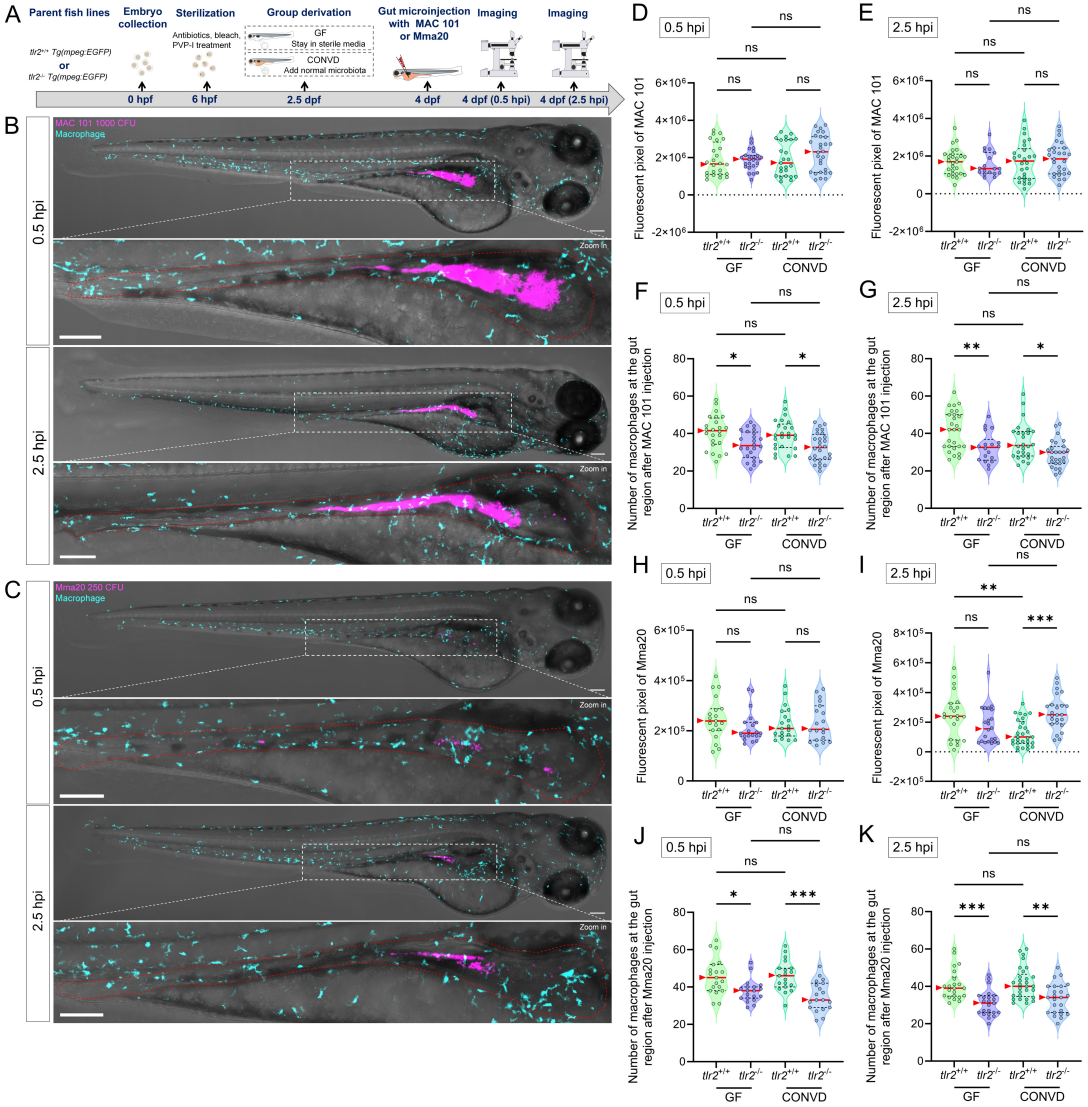
TLR2 deficiency impairs early macrophage accumulation at the gut after robotic MAC 101 and Mma20 gut microinjection. (A) Schematic of the experimental workflow. (B,C) Representative fluorescence images showing bacterial burden and macrophage number at the gut region after 0.5 and 2.5 hours of gut microinjection of MAC 101 (B) and Mma20 (C). Representative images are *tlr2* wild-type larvae under germ-free (GF) condition. Bacteria are shown in magenta. Scale bar: 100 µm for full-body images; 25 µm for zooms. (D,E) Quantification of bacterial burden after 0.5 (D) and 2.5 (E) hours of gut microinjection of MAC 101. No significant differences of bacterial burden were found among groups. (F,G) Quantification of the number of macrophages at the gut region after 0.5 (F) and 2.5 (G) hours of gut microinjection of MAC 101. The number of macrophages at the gut region was higher in the *tlr2* wild-type larvae compared to the *tlr2* mutants after 0.5 and 2.5 hours of MAC 101 gut microinjection in both GF and CONVD conditions. For MAC 101 infection, the data from GF *tlr2*^+/+^ (n=103) group, GF *tlr2*^-/-^ (n=79) group, CONVD *tlr2*^+/+^ (n=92) group and CONVD *tlr2*^-/-^ (n=110) group are based on three independent experiments. (H,I) Quantification of bacterial burden after 0.5 (H) and 2.5 (I) hours of gut microinjection of Mma20. No significant differences of bacterial burden were found among groups at 0.5 hpi. At 2.5 hpi, *tlr2* mutant larvae had higher bacterial burden than the wild-type control in the CONVD condition. For *tlr2* wild-type larvae, GF larvae had a higher bacterial burden than CONVD larvae. (J,K) Quantification of the number of macrophages at the gut region after 0.5 (J) and 2.5 (K) hours of gut microinjection of Mma20. The number of macrophages at the gut region was higher in *tlr2* wild-type larvae compared to the *tlr2* mutants after 0.5 and 2.5 hours of Mma20 gut microinjection in both GF and CONVD conditions. For Mma20 infection, the data from GF *tlr2*^+/+^ (n=79) group, GF *tlr2*^-/-^ (n=72) group, CONVD *tlr2*^+/+^ (n=59) group and CONVD *tlr2*^-/-^ (n=62) group are based on three independent experiments. Statistical significant difference was determined by one-way ANOVA, red arrows point to the median, ns, non-significant, *, *P* < 0.05, **, *P* < 0.01, ***, *P* < 0.001.

Using the gut microinjection method (site of puncture shown in Figure 3B), we monitored the short-term response of macrophages after MAC 101 and Mma20 gut injection (Figure 10A-C). We tested both GF and CONVD conditions in *tlr2* mutant and wild-type larvae. The results show that at 0.5 hours after gut injection of MAC 101 or Mma20 there was no significant difference in bacterial burden in the four groups (Figure 10D,H). At 2.5 hours after MAC 101 gut injection, still no significant difference in bacterial burden in the four groups was detected (Figure 10E). However, after 2.5 hours of Mma20 gut injection, in the CONVD condition, *tlr2* mutant larvae had higher bacterial burden than the wild-type control, while there was no significant difference in the GF condition. For *tlr2* wild-type larvae, GF larvae had a higher bacterial burden than CONVD larvae, while in *tlr2* mutant larvae there was no significant difference (Figure 10I). As presented above (Figure 3), at 24 hours after gut injection, significant values were observed in all conditions.

We also quantified the number of macrophages at the gut at 0.5 and 2.5 hours after gut microinjection (Figure 10F-G,J-K). There was no significant difference of macrophage accumulation at the gut between GF and CONVD larvae in either *tlr2* wild-type or mutant genotype at the measured time points. However, the number of macrophages at the gut region was consistently higher in *tlr2* wild-type larvae compared to the *tlr2* mutants after 0.5 and 2.5 hours of MAC 101 and Mma20 gut microinjection under both GF and CONVD conditions. (Figure 10F-G,J-K). These results show that the deficiency of *tlr2* impairs the short-term accumulation of macrophages at the gut after MAC 101 and Mma20 bacterial gut microinjection.

### 3.7. TLR2 deficiency impairs macrophage motility after MAC 101 and Mma20 gut microinjection

**Figure 11:**
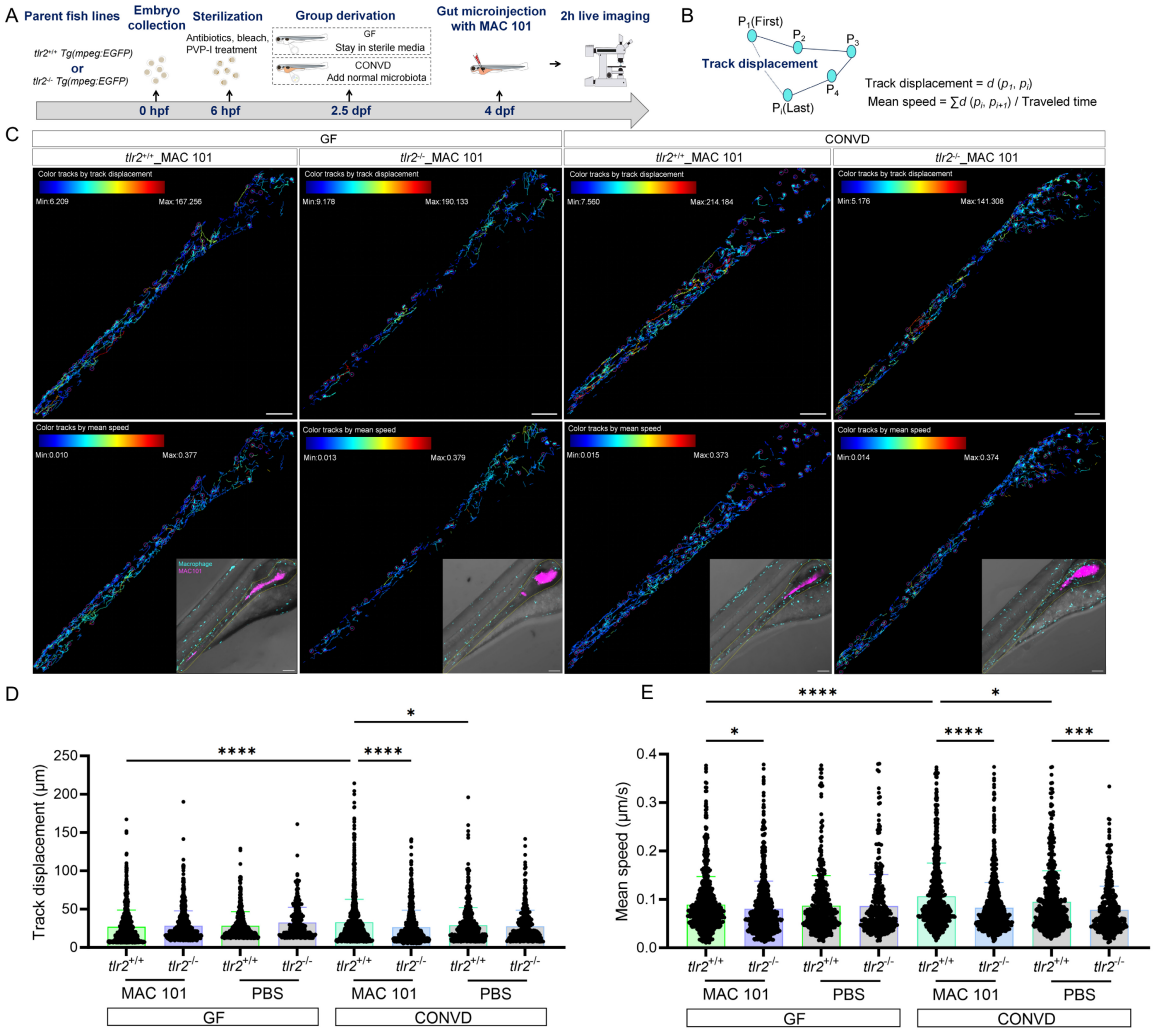
TLR2 deficiency impairs macrophage motility after robotic MAC 101 gut microinjection. (A) Schematic of the experimental workflow. (B) Calculation formulas of track displacement and mean speed. (C) Representative images showing trajectories of macrophages at the gut region in *tlr2* wild-type and mutant larvae under CONVD and GF conditions. (D) Track displacement of macrophages. (E) Mean speed of macrophages. The data from GF *tlr2*^+/+^ (n=3) group, GF *tlr2*^-/-^ (n=3) group, CONVD *tlr2*^+/+^ (n=3) group and CONVD *tlr2*^-/-^ (n=3) group are based on three independent experiments (one larva for each group per independent experiment). Statistical significant difference was determined by one-way ANOVA, red arrows point to the median, ns, non-significant, *, *P* < 0.05, ***, *P* < 0.001, ****, *P* < 0.0001.

To study the macrophage motility and behavior after MAC 101 and Mma20 gut microinjection, we first performed two hours live imaging and cell tracking analysis after robotic MAC 101 bacterial gut microinjection (Figure 11A-C). Same amount of PBS was injected to be the control group (Supplementary Figure 6). Live imaging of macrophage trajectories in the *tlr2* wild-type larvae showed that under the CONVD condition, but not under the GF condition, the track displacement and mean speed of macrophages at the gut region was larger after MAC 101 gut injection compared to gut injection with the PBS control. After MAC 101 injection, the track displacement and mean speed of macrophages at the gut region under the CONVD condition was larger than that under the GF condition in the *tlr2* wild-type larvae (Figure 11D-E). This shows that gut injection of MAC 101 triggers a higher macrophage mobility and behavior that is dependent on the microbiome. When comparing the *tlr2* wild-type larvae with the *tlr2* mutant larvae after MAC 101 gut injection, the track displacement of macrophages at the gut region was higher in the wild type under the CONVD condition. The mean speed of macrophages at the gut region in the *tlr2* wild-type larvae was larger than the mutant larvae after MAC 101 gut injection in both the GF and CONVD conditions (Figure 11D-E). These results show that *tlr2* plays a role in determining macrophage mobility and behavior after MAC 101 microinjection in the gut.

**Figure 12:**
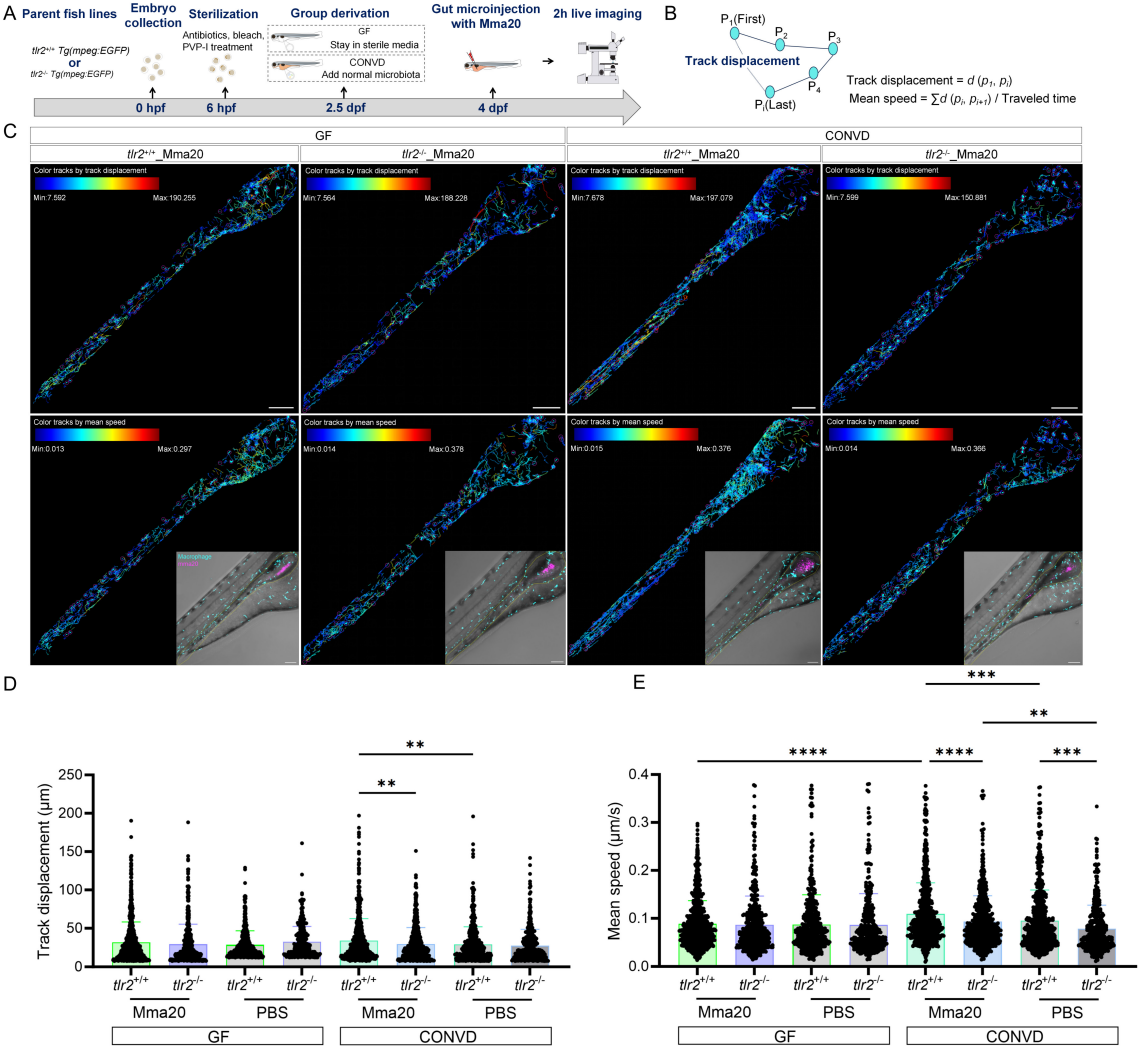
TLR2 deficiency impairs macrophage motility after robotic Mma20 gut microinjection. (A) Schematic of the experimental workflow. (B) Calculation formulas of track displacement and mean speed. (C) Representative images showing trajectories of macrophages at the gut region in *tlr2* wild-type and mutant larvae under CONVD and GF conditions. (D) Track displacement of macrophages. (E) Mean speed of macrophages. The data from GF *tlr2*^+/+^ (n=3) group, GF *tlr2*^-/-^ (n=3) group, CONVD *tlr2*^+/+^ (n=3) group and CONVD *tlr2*^-/-^ (n=3) group are based on three independent experiments (one larva for each group per independent experiment). Statistical significant difference was determined by one-way ANOVA, red arrows point to the median, ns, non-significant, **, *P* < 0.01, ***, *P* < 0.001, ****, *P* < 0.0001.

We then performed similar live imaging and cell tracking analysis after robotic Mma20 bacterial gut microinjection (Figure 12A-C). The results showed that under the CONVD condition the track displacement and mean speed of macrophages at the gut region in the *tlr2* wild-type larvae was larger after Mma20 gut injection compared to gut injection with PBS control. For *tlr2* wild-type larvae, the mean speed of macrophages at the gut region in the *tlr2* wild type under CONVD condition was larger than that under GF condition (Figure 12D-E). When comparing the *tlr2* wild-type larvae with the *tlr2* mutant larvae after Mma20 gut injection, the track displacement of macrophages at the gut region was larger in the wild type under the CONVD condition (Figure 12D). The mean speed of macrophages at the gut region in the *tlr2* wild-type larvae was larger than the mutant larvae after Mma20 gut injection in the CONVD condition (Figure 12E). These results are highly similar with the result after MAC 101 injection of the gut. Overall, these results shows that gut injection of MAC 101 and Mma20 triggers a higher macrophage mobility and behavior that is dependent on the microbiome. Importantly, the results show that *tlr2* plays a role in determining macrophage mobility and behavior after MAC 101 and Mma20 microinjection in the gut.

## 4. Discussion

### 4.1. Overview

Nontuberculous mycobacteria (NTM) infectious diseases are rising in incidence, prevalence and mortality worldwide. Although the *M. avium* subspecies *paratuberculosis* (MAP) infection in the gut of ruminants, causing Johne's disease, has been studied extensively, the role of mycobacterial infection of the human gut has been poorly documented 56, 57. Some studies have shown that mycobacteria are potential factors in human Crohn's disease, a chronic inflammatory disease of the GI tract, but a causative role of NTM in this disease or other human chronic gut diseases has not been proven 25-28. This study investigates the role of Toll-like receptor 2 (TLR2) and the microbiome in regulating immune responses in NTM infection in a zebrafish larval model. We used the natural immersion infection model and a newly developed automated gut microinjection method for studying immune responses upon infection with two NTM strains (MAC 101 and Mma20) at the genetic and cellular levels. We show that the two studied NTM strains behave differently in their proliferative properties in the gut and elicit common and specific responses at the entire organism level. Importantly, we show that TLR2 plays an important role in dissemination of the bacteria out of the gut in which process the macrophages serve as key mediators.

### 4.2. Mycobacterial localization, dissemination, and transcriptional responses are modulated by TLR2 and the microbiome

Our results reveal distinct proliferation rates and spatial colonization patterns of MAC 101 and Mma20 in the zebrafish gut after immersion infection and gut microinjection. After immersion infection, MAC 101 preferentially localized to the posterior part of the gut and Mma20 to the anterior gut in wild-type microbiome-colonized larvae (Figure 1D,J). These results show unexpected species-specific localization patterns of MAC 101 and Mma20 in the gut that are currently unexplainable*.* While these imaging data reveal strain-dependent spatial distributions at the body-region level, future studies with detailed histological characterization, including hematoxylin and eosin (H&E) and Ziehl-Neelsen staining of defined intestinal regions, will be required to resolve intestinal mucosal localization and associated pathology. Such follow up studies can make use of the recently published methods for ultrastructural mapping of the zebrafish larval gastrointestinal system at later stages than our study 58. It is interesting that in the absence of *tlr2* or a microbiome there is no difference anymore in preference for the distribution in the gut for the two bacterial strains. This shows that both TLR2 and the microbiome influence the shown spatial bacterial distribution for both MAC 101 and Mma20 bacteria in the gut. We note that the observed anterior/posterior enrichment should not be interpreted as a fixed, static localization. Instead, the regional bias likely reflects dynamic processes in the gut, potentially including differences in luminal flow, gut peristalsis, mucus properties, local immune activity, and microbiome-dependent factors that influence bacterial retention and transit. Future time-course experiments and functional assays will be needed to define the temporal dynamics of this regional bias and to test whether it persists during longer infections.

Considering that *M. avium* is of clinical relevance for gut infection, it is of interest to further study the determinants of specificity of mycobacteria for their localization. This can be predicted to be based on a high complex signaling interplay between the microbes and their host which involves via the TLR2 pathway 38. At the whole organism gene expression level, we can already conclude from our qRT-PCR analyses that there are distinct differences of the host response to the two NTM strains that are dependent on the microbiome. Considering that the chosen markers *fosl1a* and *cebpb* are downstream of TLR2 signaling, but still exhibit marked differences in their responses, we envisage that the regulatory mechanisms underlying specific responses also includes other factors 38. Factors that come to mind are other TLRs and intermediators such as factors from the mucus like uromodulin, Glycoprotein 2 (GP2) and omcins that have been studied in the zebrafish model, but are also relevant for mammalian microbiome studies 38, 59.

### 4.3. Macrophages contribute to the phagocytosis and dissemination of mycobacteria to posterior body region

After immersion infection with MAC 101 and Mma20, bacteria are found not only in the gut region, but also in other tissues outside the gut and even posterior body region such as the tail. To examine the oral route of bacterial entry, we performed immersion infection with larvae in which the head was immersed in agarose to block bacterial entrance and we show that MAC 101 and Mma20 bacteria are unable to invade the body through undamaged intact skin. This finding aligns with previous reports that cutaneous diseases causing by mycobacterial skin infections usually result from hematogenous dissemination or spread from underlying wounding foci 60. Furthermore, gill infections are rarely found in our immersion infectious model and gill-infected individuals have been excluded from data analysis. Therefore, after excluding bacterial entry from the skin or gills, we infer that bacteria disseminated in other body tissues come from the gut. This conclusion is further supported by our gut microinjection experiments, which demonstrate that direct introduction of bacteria into the gut can result in efficient dissemination to posterior body region, confirming the gut as the primary source of systemic bacterial spread after oral entrance.

How mycobacteria disseminate from the gut, particularly to posterior body region, remains an important question in understanding their pathogenesis. From our imaging data, both MAC 101 and Mma20 bacteria located in posterior body region are most frequently found within macrophages (Figure 7B, Figure 8B). Therefore, one possibility for the mechanism of bacterial entrance is that macrophages phagocytose bacteria in or close to the lining of the gut and act as carriers, transporting the intracellular pathogens to distant sites through the body. This transport function of macrophages has been demonstrated in other mycobacterial infections in mice and zebrafish model that were introduced through injection 61. In our macrophage ablation experiments, this transporting role of macrophages is also confirmed by the observation that there is almost no bacterial dissemination to the posterior body region in macrophage ablated larvae (Figure 5). We note, however, that our imaging-based readout reflects spatial redistribution at the body-region level and does not resolve colonization of specific organs or tissue compartments. Future studies using serial histology, including Ziehl-Neelsen staining, will be required to identify the affected organs and to localize bacteria within defined tissue microenvironments. Also, fluorescent signal of bacteria reports bacterial localization and detection and does not by itself distinguish live from dead bacteria. Future studies combining fluorescence imaging with dedicated viability readouts and histology will be required to directly test microenvironment-dependent effects on bacterial viability.

Studies have shown that *M. tuberculosis* and *M. marinum* bacteria preferentially recruit and infect permissive macrophages while evading microbicidal ones. One of the host strategies to cope with this immune evasion is to recruit microbicidal macrophages through TLR2-dependent signaling 61. From our results, TLR2 deficiency leads to a significant reduction in the number of macrophages containing MAC 101 and Mma20 bacteria (Figure 5-8). These findings demonstrate the important role of TLR2 in mediating the function of macrophages after infection with mycobacteria. For future studies, employing robust systems to determine whether macrophages which contain mycobacteria are permissive or microbicidal will help to further understand the mechanisms. Advanced tools such as single-cell analyses and electron microscopy (EM) studies can provide critical insights into host-pathogen interactions at the cellular and ultrastructural levels in the gut tissue and can better understand the role that macrophages play in this process.

### 4.4. Macrophage behavior and motility after mycobacterial infection are regulated by TLR2 and the microbiome

The gut mucosal immune system serves as a critical barrier, protecting the host from the invasion of infectious pathogens and facilitating the elimination of harmful non-self antigens 62. In the elimination process, macrophages in the gut tissue are among the first immune cells mycobacteria encounter 63. By using the mycobacteria gut microinjection technique, we further study the macrophage behavior after directly injecting MAC 101 and Mma20 bacteria to the gut lumen. TLR2 mutants display reduced macrophage accumulation at the gut and decreased macrophage motility after MAC 101 and Mma20 gut microinjection. TLR2 has been shown to regulate macrophage migration behavior in the mycobacterial zebrafish infection model using microinjection into the tail fin 15. This study demonstrated that TLR2 has a function in the mechanisms that control migration speed of macrophages to infection sites upon *M. marinum* infection 15. Our current findings show that TLR2 facilitates the recruitment of macrophages to the mycobacterial infection sites during early infection via the gut tissues. These findings provide evidence for the responses of macrophages to mycobacteria that enter via a natural infection route are also dependent on TLR2 signaling.

Using our bacterial gut microinjection system, the results show that TLR2 deficiency leads to a diminished mean speed of macrophages at the gut after injection with PBS control, suggesting that TLR2 has a role in wounding responses and this finding is in agreement with the results of our previous tail fin wounding model in zebrafish larvae 46. However, the regulatory role of TLR2 in wounding responses is only shown when the larvae are colonized by a microbiome. After MAC 101 and Mma20 gut microinjection, the larvae under the normal microbiome-colonized condition present a higher mean speed of macrophages compared with larvae under the germ-free condition for the wild-type larvae, but not this case in the *tlr2* mutant larvae (Figure 10-11). These results suggest that TLR2 signaling is required for microbiome-mediated modulation of innate immune cell dynamics at the systems level. Previous studies have shown that *M. tuberculosis* initiates infection in the relatively sterile environment of the lower respiratory tract, rather than in the upper respiratory tract, where resident microflora and inhaled environmental microbes 61. One perspective is that the presence of these microbiota results in the recruitment of microbicidal macrophages through TLR-dependent signaling 61. Our findings support this perspective and extend it to the intestinal context, highlighting a potential mechanism by which the microbiome, through TLR2 signaling, modulates macrophage behavior to influence the responses of mycobacterial infection.

We have chosen the *mmp9* gene as a marker for studying gene transcription response to gut infection since it is an obvious candidate to trigger response of macrophages as shown for studies of granuloma formation in response to *M. marinum* infection 55. Our results show that* mmp9* induction by after infection by MAC 101 and Mma20 is dependent on TLR2, although it is not essential (Figure 4). Analogous to the results of Volkman et al, it can be envisaged that macrophages are attracted to the epithelial cells of the gut due to secretion of MMP9 protein that is dependent on TLR2 55. This might also involve other TLRs such as TLR8, that has been linked to TLR2 signaling by Hu et al 21. Further studies will need detailed single cell transcriptome studies to unravel the complex interactions between MAC 101 and Mma20 with the gut. However, it will be difficult to understand the complexity in a natural system since it is still very difficult to analyze the transcriptome response of the bacteria to the host and the other bacteria in the microbiome. Hopefully single bacterial *in situ* transcriptome analyses tools that are rapidly developing could be employed in the future for analysis in the zebrafish gnotobiotic system 64-66.

### 4.5. Potential translational role of TLR2 and the microbiome in the mycobacterial gut disease

The recognition of mycobacteria such as *M. tuberculosis* by macrophage PRRs is primarily mediated by TLR2 3, 21, 67. Due to its crucial role in orchestrating the host immune response, TLR2 has been extensively studied as a potential therapeutic target for mycobacterial infectious diseases, with most research focusing on pulmonary infections 68-71. For instance, enhancing TLR2 signaling has been shown to restore immune responses and ameliorate susceptibility to NTM lung disease 71. In our study, TLR2-deficient zebrafish larvae present reduced macrophage phagocytic capacity and impaired macrophage migration upon NTM infection. These findings indicate a protective role of TLR2 in mycobacterial gut infections, which is consistent with its known function in lung infection models.

The microbiome plays a pivotal role in shaping host immunity and affecting susceptibility to gut infectious diseases 39, 40, 72. We are therefore interested in the role of the microbiome in mycobacterial gut infections. Several studies have explored the microbiome's impact on mycobacterial pulmonary infections, for example showing a correlation between gut microbiota dysbiosis and progression of disease in patients with NTM lung infections 71, 73, 74. However, its role in mycobacterial gut infections remains under-investigated. In this study, we demonstrate that the presence or absence of a microbiome significantly influences bacterial localization, host transcriptional responses, and macrophage behavior after MAC 101 and Mma20 gut infections. Larvae reared under GF conditions exhibited higher bacterial colonization *in vivo* and reduced macrophage motility compared to conventionally colonized counterparts, highlighting the microbiome's contribution to immune modulation. Notably, these effects were dependent on TLR2 signaling, suggesting a TLR2-microbiome axis that regulates host defense against mycobacterial gut infection. A function of TLR2 in controlling inflammatory responses at the systems level has also been shown in a gnotobiotic zebrafish larval model in the absence of pathogenic infection 38.

A study combining Crohn's disease patient data with an *in vitro* Caco-2 epithelial model showed that enteropathogenic infection can modulate serotonin transporter (SERT) function via TLR2-dependent signaling, which supports the important role of TLR2 in human gut infectious disease 75. The role of TLR2 in mycobacterial gut infections has not been studied in a human gut disease setting, but it has been studied in various ruminants suffering from Johne's disease and in gut mammalian cell cultures using genetic and *in situ* approaches 24, 75-77. For instance, the TLR2 haplotypes encoding the polymorphism Q650 have been shown to facilitate selective breeding for Turkish sheep with reduced susceptibility to Johne's disease 24. In a bovine paratuberculosis study, the number of TLR2- expressing macrophages was found to be correlated with the quantity and focal localization of bacteria in neighboring infected lesions 76. In addition, TLR2 signaling has been shown to play a role in phagosome trafficking and antimicrobial responses in cultured bovine mononuclear phagocytes infected with *M. avium* subsp. *Paratuberculosis* 77.

Several papers have highlighted the pathological similarities between Johne's disease and Crohn's disease 78, 79. Crohn's disease is a chronic, nonspecific inflammatory bowel disease (IBD) of the intestine. Its cause is undetermined, but it is widely accepted to result from a complex interplay of genetic susceptibility, immune dysregulation, environmental factors, and intestinal microbiota 80. Some studies have suggested a potential role of *M. avium* subsp. *paratuberculosis* in Crohn's disease pathogenesis due to its detection in intestinal lesions of patients and its pathological similarities to Johne's disease in ruminants 81. Crohn's disease is also associated with gut microbiome dysbiosis, which disrupts the balance of immune tolerance and promotes persistent activation of mucosal immune cells, contributing to chronic inflammation 82. There are many histochemical, genetic and functional studies that show a role of TLR2 in Crohn's disease 81, 83-86. This is in line with the important functions of TLR2 in controlling gut homeostasis, disease and epithelial tissue repair in a complex interplay with the microbiome 22. Our zebrafish infection model offers a valuable platform to explore a putative causative role of mycobacteria in Crohn's disease. In addition, it can help to distinguish responses to infection and tissue damage as we have shown previously in a zebrafish tail wounding model 46. The model's capacity for high-throughput infection, live imaging, and genetic manipulation enables detailed investigation of host-pathogen interactions, immune signaling, and microbiome dynamics *in vivo*. In this study, interindividual variation was evident across infection experiments, which is an inherent feature of whole-organism larval zebrafish models and likely reflects biological heterogeneity in immune responses, microbiome composition, bacterial exposure, and early colonization dynamics. Small differences in developmental stage, innate immune cell distribution, or initial uptake into the gastrointestinal track may therefore lead to divergent dissemination trajectories among individuals. Accordingly, our conclusions are based on statistically supported population-level trends across multiple independent experiments, while acknowledging that individual larvae can exhibit distinct infection outcomes.

The specific effects of the TLR2 chemical inhibitor C29 in our zebrafish model, recapitulating the results of a TLR2 mutant, shows that further chemical screening could lead to potential medicines that limit the dangers of dissemination of mycobacteria at early stages of infectious disease in the gut. Consistent with this translational direction, the TLR2 antagonist CU-CPT22 has been shown to inhibit Raw 264.7 macrophage migration toward MAC 101-infected mouse gut organoids, providing a mammalian parallel to the TLR2-dependent macrophage migratory response observed in our zebrafish model 87. Complementing the *in vivo* zebrafish model, future *in vitro* experiments in human immune cells will be valuable to further define the mechanistic function of TLR2 in the migration of macrophages underlying mycobacterial dissemination.

## 5. Conclusions

In conclusion, our results reveal distinct proliferation rates and spatial colonization patterns of the nontuberculous mycobacteria MAC 101 and Mma20 in the zebrafish gut after immersion infection and gut microinjection. We demonstrate that TLR2 and the microbiome play critical roles in modulating host responses to MAC 101 and Mma20 gut infection. Specifically, the dissemination of bacteria by macrophages after immersion infection depends on TLR2, but not on the microbiome. Macrophage motility after robotic bacterial gut microinjection was impaired in *tlr2* mutant larvae compared to the wild type. However, the presence of a microbiome significantly influences bacterial localization, host transcriptional responses, and macrophage behavior in a TLR2-dependent manner. These findings highlight a TLR2-microbiome axis as a key regulator of innate immunity during mycobacterial gut infection. Our results underscore the importance of microbiota-derived signals in shaping immune defense and suggest that targeting TLR2 signaling or microbiome composition could be a potential strategy for managing mycobacterial gut diseases. Further investigation in mammalian systems and clinical studies will help to validate and extend these insights to human intestinal diseases.

## Supplementary Material

Supplementary figures and tables, video legends.

Supplementary video 1.

Supplementary video 2.

Supplementary video 3.

## Data Availability

The data obtained from this study is available upon request.
