# Supplementary material for: The function of TLR2 and the microbiome in macrophage-dependent dissemination of nontuberculous mycobacterial gut infection
Source: Int J Biol Sci. 2026 May 18;22(10):5359–84. doi: 10.7150/ijbs.124776 (PMC13215361; doi:10.7150/ijbs.124776)
Supplement: Supplementary file 1 — Supplementary figures and tables, video legends. [file ijbsv22p5359s1.pdf]

Table S1. The qRT-PCR primers used in this study

Primers	Sequences	Application
il1b-F	5'...TGTGTGTTTGGGAATCTCCA...3'	qRT-PCR
il1b-R	5'...CTGATAAACCAACCGGGACA...3'	qRT-PCR
mmp9-F	5'...CATTAAAGATGCCCTGATGTATCCC...3'	qRT-PCR
mmp9-R	5'...AGTGGTGGTCCGTGGTTGAG...3'	qRT-PCR
cebpb-F	5'...GCAGGCAACCTATCACCTACATAC...3'	qRT-PCR
cebpb-R	5'...CGCAAGTTTCACCGACTACAAGT...3'	qRT-PCR
fosl1a-F	5'...CTCAGCCCTCCCAATCACATCT...3'	qRT-PCR
fosl1a-R	5'...TACACTTCGCCGCAGCCATT...3'	qRT-PCR
ppial-F	5'...ACACTGAAACACGGAGGCAAAG...3'	Reference
ppial-R	5'...CATCCACAACCTTCCCGAACAC...3'	Reference

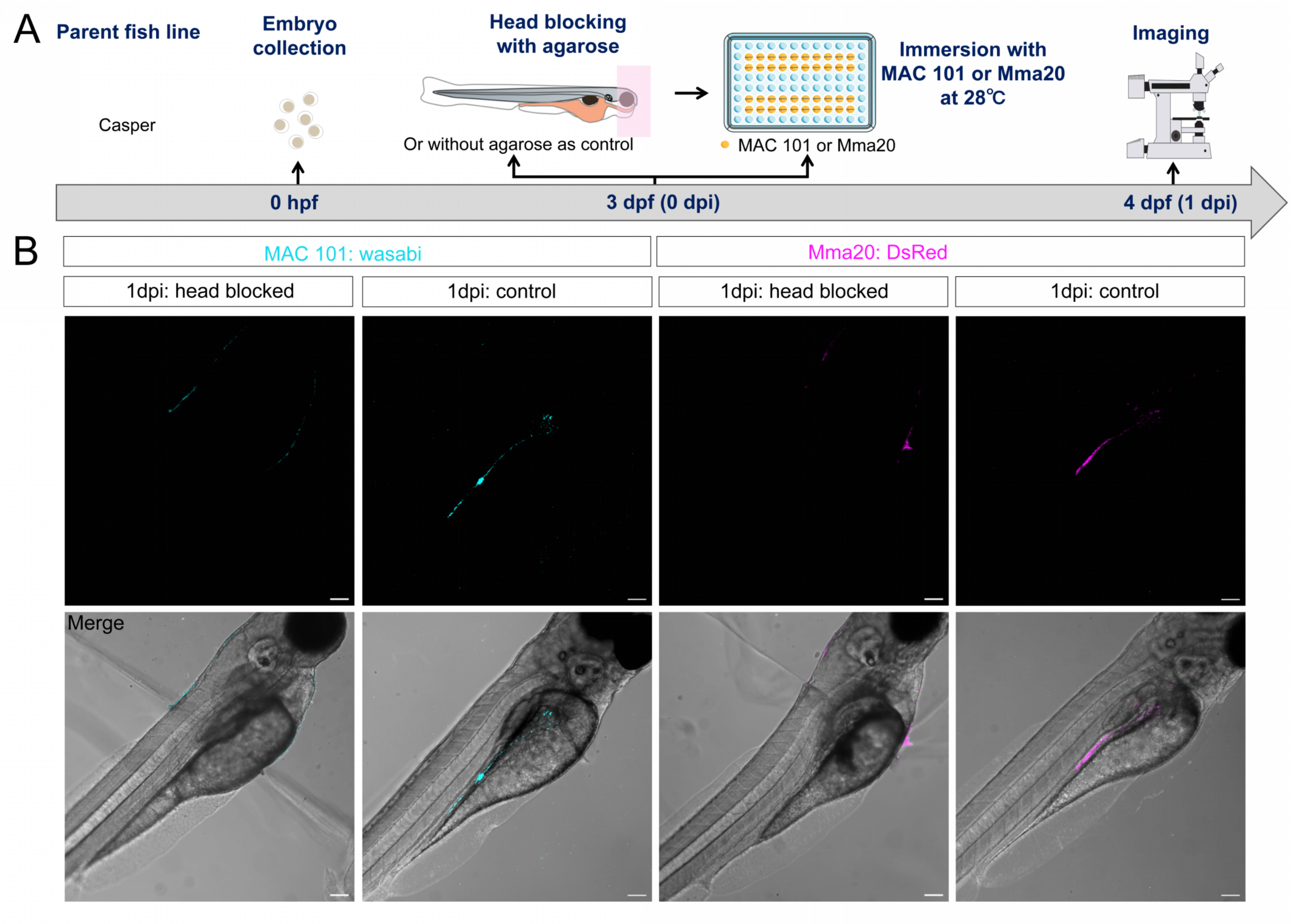

Supplementary figure 1. Natural immersion inoculation with MAC 101 and Mma20 after the head is blocked with agarose. (A) Schematic of the experimental workflow. (B) Representative fluorescence images showing that MAC 101 and Mma20 bacteria do not colonize internal tissues when the head is physically blocked during immersion infection. Scale bar: 100 µm.

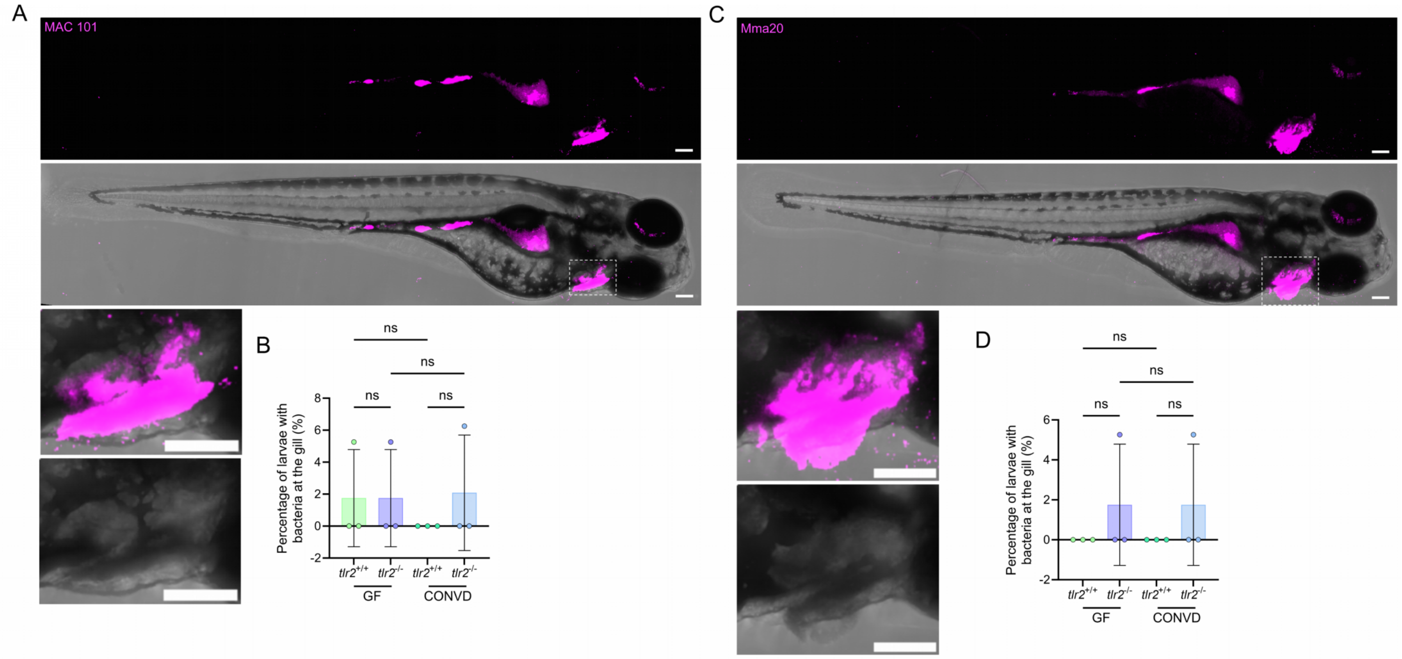

Supplementary figure 2. Larvae with gill infection after immersion with MAC 101 and Mma20. (A) Representative fluorescence images of MAC 101 gill infection. (B) Percentage of larvae with gill infection after immersion with MAC 101. (C) Representative fluorescence images of Mma20 gill infection. (D) Percentage of larvae with gill infection after immersion with Mma20. Scale bar: 100 μm for full-body images; 25 μm for zooms. ns, non-significant.

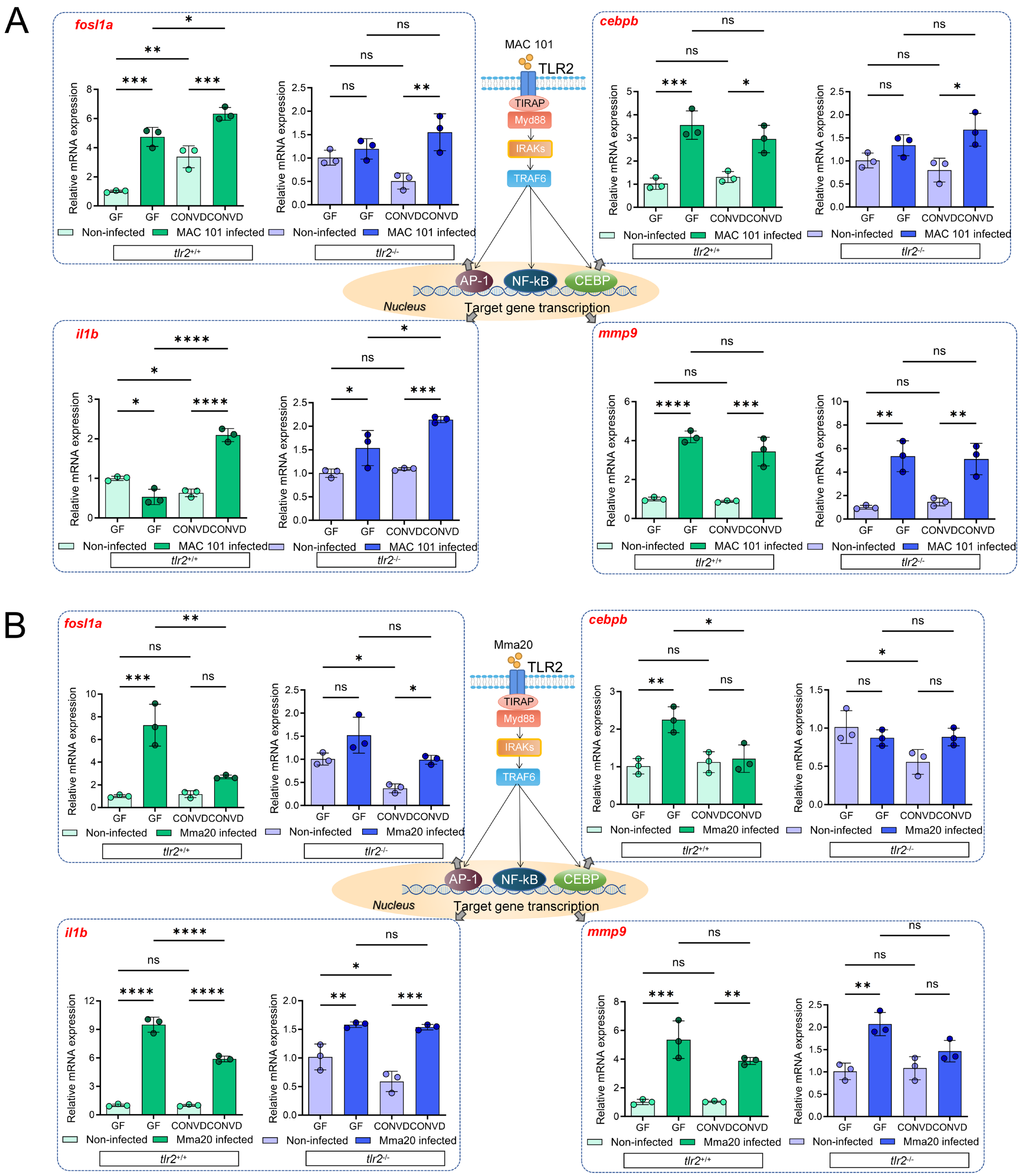

Supplementary figure 3. Immune-related gene expression profiles after MAC 101 and Mma20 immersion infection. (A) Immune-related gene expression profiles after MAC 101 immersion infection. (B) Immune-related gene expression profiles after Mma20 immersion infection. Statistical significant difference was determined by one-way ANOVA, ns, non-significant, *, $P < 0.05$, **, $P < 0.01$, ***, $P < 0.001$, ****, $P < 0.0001$.

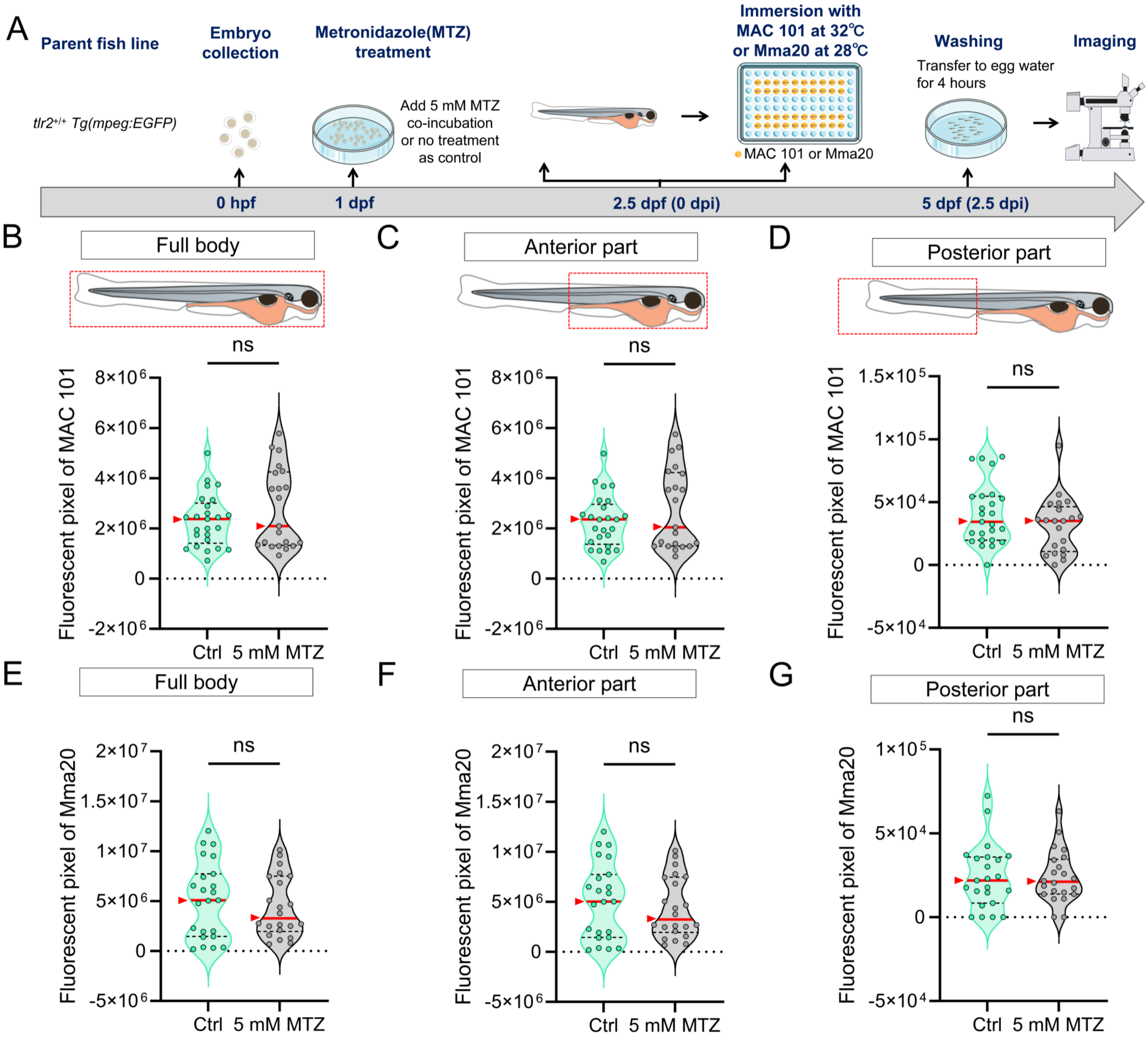

Supplementary figure 4. Treatment of 5 mM metronidazole (MTZ) has no effect on MAC 101 and Mma20 infection on zebrafish larvae. (A) Schematic of the experimental workflow. Zebrafish larvae were treated with 5 mM metronidazole (MTZ) at 1 dpf and no treatment as the control group. Larvae from these two groups were immersed with mCherry-labeled MAC 101 or DsRed-labeled Mma20 at 2.5 dpf for 2.5dpi. (B-D) Quantification of the bacterial burden in the full body (B), anterior part of the body (C) and posterior part of the body (D) after infection with MAC 101. For MAC 101 infection, the data from control group (n=27) and 5 mM MTZ treatment group (n=21) are based on two independent experiments. (E-G) Quantification of the bacterial burden in the full body (E), anterior part of the body (F) and posterior part of the body (G) after infection with Mma20. For Mma20 infection, the data from control group (n=23) and 5 mM MTZ treatment group (n=22) are based on two independent experiments.

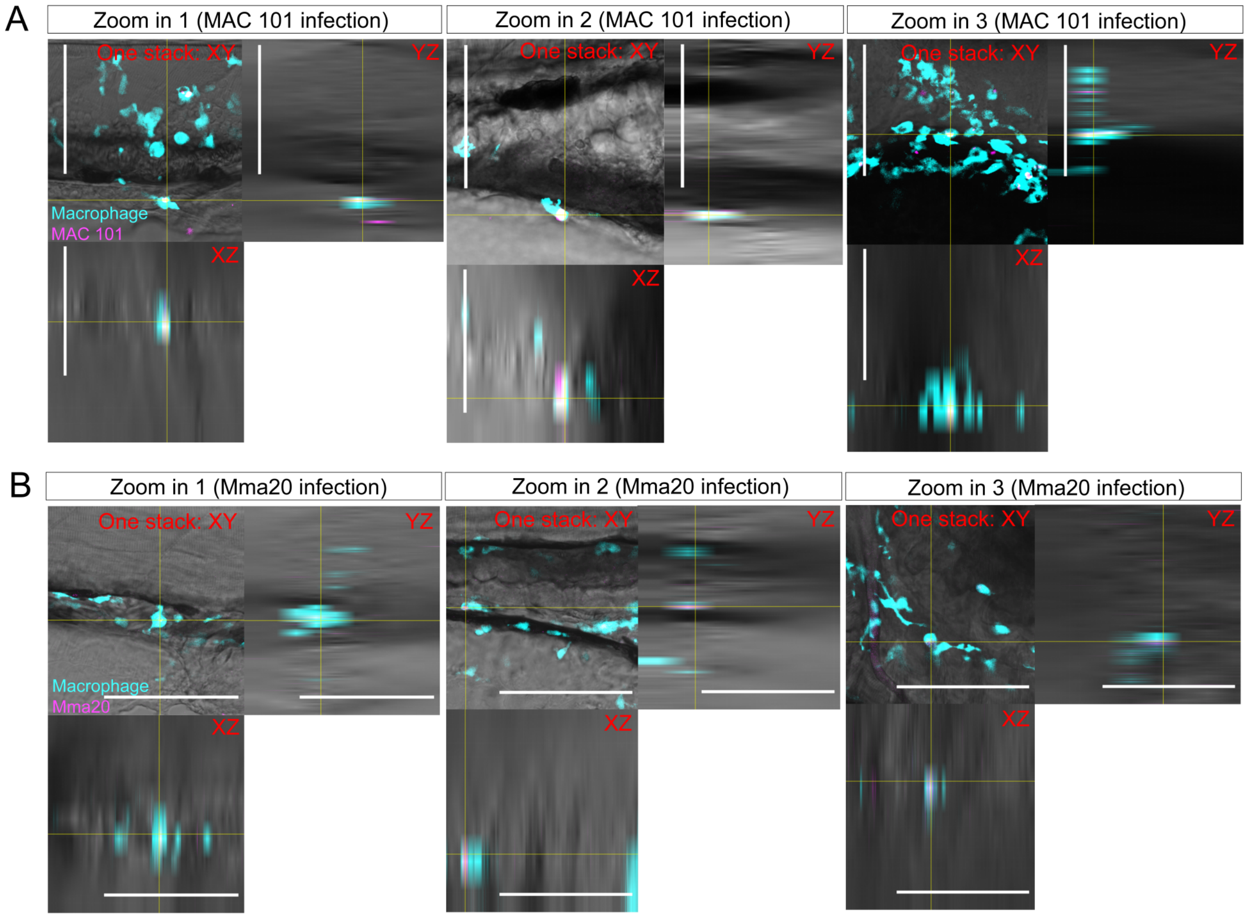

Supplementary figure 5. Orthogonal views (XY, XZ, and YZ) of confocal image stacks of zebrafish larvae after natural immersion infection with MAC 101 (A) and Mma20 (B). Bacteria are shown in magenta. Macrophages are shown in cyan. Scale bar: 100 µm.

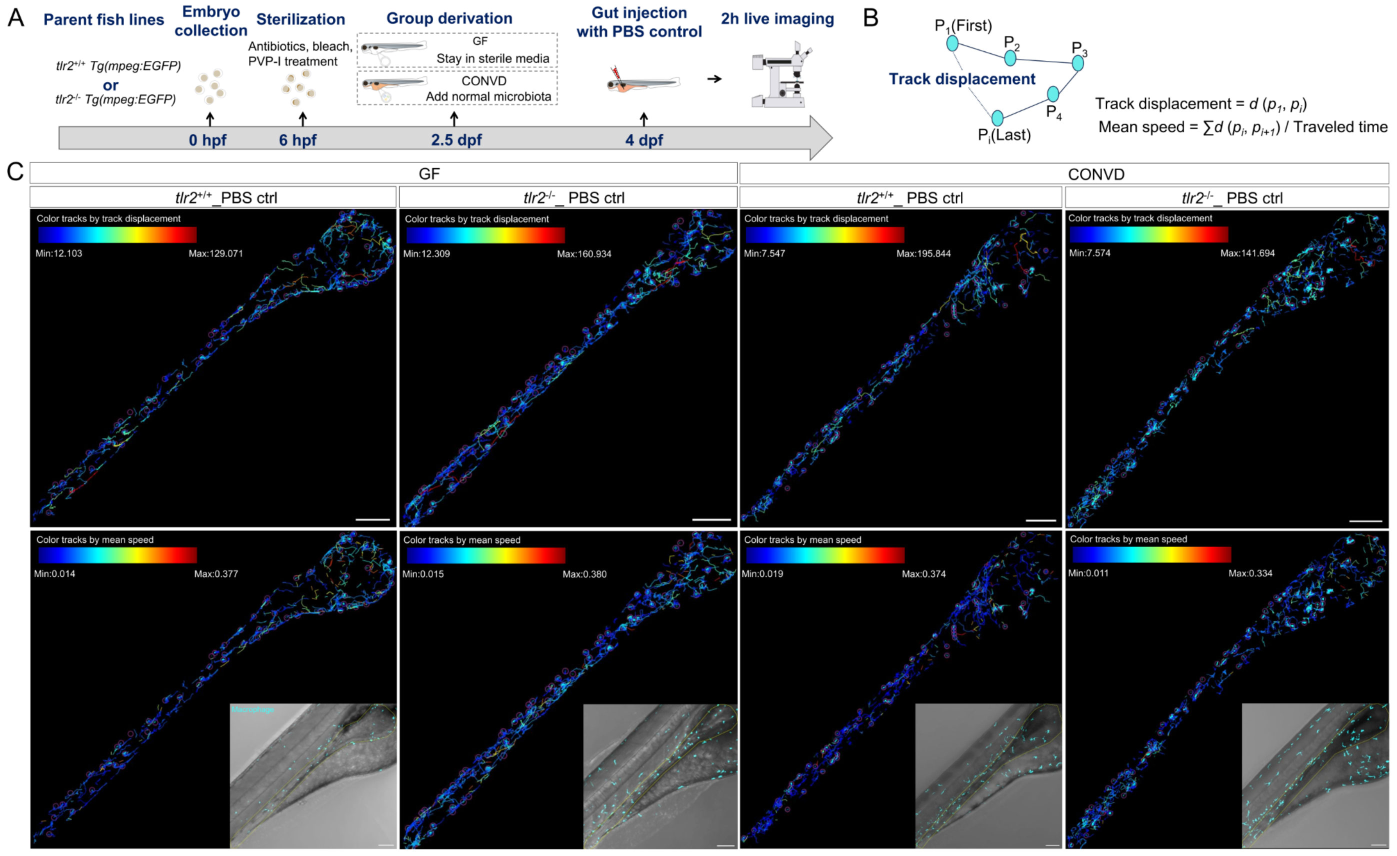

Supplementary figure 6. Macrophage motility at the gut after robotic gut microinjection with PBS control. (A) Schematic of the experimental workflow. (B) Calculation formulas of track displacement and mean speed. (C) Representative images showing trajectories of macrophages at the gut region in *tlr2* wild-type and mutant larvae under CONVD and GF conditions. (D) Track displacement of macrophages. (E) Mean speed of macrophages.

Supplementary video 1. Larva after head blocked with agarose is still alive. (Please double click the video to play.)

Supplementary video 1.avi

Supplementary video 2. Robotic gut bacterial microinjection process using the automated microinjection system for zebrafish larvae. (Please double click the video to play.)

Supplementary video 2.mp4

Supplementary video 3. Migration of macrophages infected with mycobacteria. (Please double click the video to play.) Bacteria are shown in magenta. Macrophages are shown in cyan. Scale bar: 100 μm.

Supplementary video 3.mp4
